# Effect of Tree Nuts on Glycemic Control in Diabetes: A Systematic Review and Meta-Analysis of Randomized Controlled Dietary Trials

**DOI:** 10.1371/journal.pone.0103376

**Published:** 2014-07-30

**Authors:** Effie Viguiliouk, Cyril W. C. Kendall, Sonia Blanco Mejia, Adrian I. Cozma, Vanessa Ha, Arash Mirrahimi, Viranda H. Jayalath, Livia S. A. Augustin, Laura Chiavaroli, Lawrence A. Leiter, Russell J. de Souza, David J. A. Jenkins, John L. Sievenpiper

**Affiliations:** 1 Toronto 3D Knowledge Synthesis and Clinical Trials Unit, Clinical Nutrition and Risk Factor Modification Center, St. Michael’s Hospital, Toronto, Ontario, Canada; 2 Department of Nutritional Sciences, Faculty of Medicine, University of Toronto, Toronto, Ontario, Canada; 3 Department of Pathology and Molecular Medicine, Faculty of Health Sciences, McMaster University, Hamilton, Ontario, Canada; 4 Division of Endocrinology and Metabolism, St. Michael’s Hospital, Toronto, Ontario, Canada; 5 Li Ka Shing Knowledge Institute, St. Michael’s Hospital, Toronto, Ontario, Canada; 6 College of Pharmacy and Nutrition, University of Saskatchewan, Saskatoon, Saskatchewan, Canada; 7 Department of Clinical Epidemiology & Biostatistics, Faculty of Health Sciences, McMaster University, Hamilton, Ontario, Canada; 8 School of Medicine, Faculty of Health Sciences, Queen’s University, Kingston, Ontario, Canada; 9 Department of Human Health and Nutritional Sciences, College of Biological Sciences, University of Guelph, Guelph, Ontario, Canada; CUNY, United States of America

## Abstract

**Background:**

Tree nut consumption has been associated with reduced diabetes risk, however, results from randomized trials on glycemic control have been inconsistent.

**Objective:**

To provide better evidence for diabetes guidelines development, we conducted a systematic review and meta-analysis of randomized controlled trials to assess the effects of tree nuts on markers of glycemic control in individuals with diabetes.

**Data Sources:**

MEDLINE, EMBASE, CINAHL, and Cochrane databases through 6 April 2014.

**Study Selection:**

Randomized controlled trials ≥3 weeks conducted in individuals with diabetes that compare the effect of diets emphasizing tree nuts to isocaloric diets without tree nuts on HbA1c, fasting glucose, fasting insulin, and HOMA-IR.

**Data Extraction and Synthesis:**

Two independent reviewer’s extracted relevant data and assessed study quality and risk of bias. Data were pooled by the generic inverse variance method and expressed as mean differences (MD) with 95% CI’s. Heterogeneity was assessed (Cochran Q-statistic) and quantified (I^2^).

**Results:**

Twelve trials (n = 450) were included. Diets emphasizing tree nuts at a median dose of 56 g/d significantly lowered HbA1c (MD = −0.07% [95% CI:−0.10, −0.03%]; P = 0.0003) and fasting glucose (MD = −0.15 mmol/L [95% CI: −0.27, −0.02 mmol/L]; P = 0.03) compared with control diets. No significant treatment effects were observed for fasting insulin and HOMA-IR, however the direction of effect favoured tree nuts.

**Limitations:**

Majority of trials were of short duration and poor quality.

**Conclusions:**

Pooled analyses show that tree nuts improve glycemic control in individuals with type 2 diabetes, supporting their inclusion in a healthy diet. Owing to the uncertainties in our analyses there is a need for longer, higher quality trials with a focus on using nuts to displace high-glycemic index carbohydrates.

**Trial Registration:**

ClinicalTrials.gov NCT01630980

## Introduction

Tree nuts are a healthy source of vegetable protein [1], unsaturated fatty acids [1], [2], fibre [3], antioxidants [4], vitamins (i.e. folic acid, vitamin B6, niacin, tocopherols), minerals (i.e. magnesium, potassium, calcium), and phytochemicals (i.e. phytosterols) [5]. Over the last two decades, a large body of evidence regarding tree nut consumption and related health outcomes has emerged from both epidemiological and controlled trials [1]. A recent large pooled analysis of two of the Harvard cohorts, as well as a recent meta-analysis of prospective cohort studies support an all-cause mortality benefit [6], [7]. There is also strong evidence that tree nuts lower LDL-cholesterol [8], which has resulted in an FDA qualified health claim [9] and their inclusion in heart association guidelines for cardiovascular risk reduction [10], [11].

The data for diabetes related outcomes have not been as consistent. Although some cohort studies show that frequent nut consumption is associated with lower incidence of type 2 diabetes [12], [13], other cohort studies do not [14], [15]. In addition, 2 recent systematic review and meta-analysis of prospective cohort studies showed no overall significant association between nut consumption and type 2 diabetes risk [7], [16], which was consistent with a subgroup analysis of the PREDIMED trial that showed a non-significant reduction in diabetes risk for individuals consuming a Mediterranean diet supplemented with nuts in comparison to a low fat diet [17]. There has been relatively few controlled trials that have specifically investigated the effects of tree nuts on glycemic control. Despite coronary heart disease being a major cause of death in individuals with diabetes, consumption of tree nuts alone have not been included as part of the recommendations in most diabetes guidelines [18]–[20], with the exception of their recent inclusion as part of various dietary/eating patterns (DASH, Mediterranean, vegetarian and vegan, and low carbohydrate diets) in American (ADA) and Canadian (CDA) diabetes association clinical practice guidelines [19], [20].

Primary prevention and management of diabetes through diet and lifestyle modification remains the cornerstone of therapy [21], [22]. In order to provide better evidence-based guidance on the role of tree nuts on glycemic control, a systematic review and meta-analysis of randomized controlled dietary trials was performed to assess the effect of tree nuts under isocaloric conditions on the endpoints HbA1c, fasting glucose, fasting insulin, and homeostasis model assessment of insulin resistance (HOMA-IR) in individuals with diabetes. The primary outcome and measurement of this study consists of a pooled analysis for each glycemic endpoint.

## Methods

The *Cochrane Handbook for Systematic Reviews of Interventions* was followed for the planning and conduct of this meta-analysis [23]. Reporting followed the Preferred Reporting Items for Systematic Reviews and Meta-Analyses (PRISMA) guidelines [24]. The review protocol is available at ClinicalTrials.gov (registration number: NCT01630980).

### Data Sources and Searches

We searched the databases MEDLINE, EMBASE, CINAHL, and the Cochrane Central Register of Controlled Trials through 6 April 2014 using the search strategy shown in **Table S1**. Manual searches of references also supplemented the electronic search.

### Study Selection

We included randomized controlled dietary trials that compared a diet emphasizing intake of tree nuts (almonds, Brazil nuts, cashews, hazelnuts, macadamia nuts, pecans, pine nuts, pistachios and walnuts) [25] on HbA1c, fasting glucose, fasting insulin, and HOMA-IR in comparison to diets without tree nuts matched for energy (isocaloric) for a follow-up period ≥3 weeks in people with diabetes. Trials that consisted of a non-randomized treatment allocation, <3 weeks of follow-up duration, non-isocaloric comparisons, lacked a suitable control, were not conducted in individuals with diabetes, or did not provide suitable endpoint data were excluded. No restrictions were placed on language.

### Data Extraction and Quality Assessment

Two investigators (EV and SB) independently reviewed all reports that met the inclusion criteria. A standardized form was used to extract relevant information on sample size, subject characteristics (health status, gender, age, weight, etc.), study setting, study design, level of feeding control, nut dose, nut type and form (whole or meal form), comparator, macronutrient breakdown of background diet(s), energy balance, follow-up duration, and funding source. The mean ± SD values were extracted for HbA1c, fasting glucose, fasting insulin, and HOMA-IR. Trials that did not report SD’s were derived from available data (95% CI, P-values, *t* or *F* statistics, SE) using standard formulae [23].

The quality of each trial was assessed using the Heyland Methodological Quality Score (MQS) where a maximum score of 13 points could be received on the basis of the trials methods, sample, and intervention [26]. Trials receiving scores of ≥8 were considered to be of higher quality. Disagreements on Heyland MQS scores were reconciled by consensus. Study quality was not assessed for those trials reported exclusively in a published abstract.

Trials were assessed for risk of bias using the Cochrane Risk of Bias Tool [23]. Domains of bias assessed were sequence generation, allocation concealment, blinding, outcome data, and outcome reporting. Trials were marked as high risk of bias when the methodological flaw was likely to have affected the true outcome, low risk of bias if the flaw was deemed inconsequential to the true outcome, and unclear risk of bias when insufficient information was provided to permit judgment. All disagreements were resolved by consensus. Authors were contacted for additional information where necessary [27]–[30].

### Data Synthesis and Analysis

Data were analyzed using Review Manager (RevMan), version 5.2 (The Nordic Cochrane Centre, The Cochrane Collaboration, Copenhagen, Denmark) for primary analyses. The difference between the intervention and control arm’s change from baseline value was derived from each trial for the endpoints HbA1c, fasting glucose, fasting insulin, and HOMA-IR. If change from baseline values were not available, end-of-treatment values were used. For trials containing multiple intervention or control arms a weighted average was applied to combine them in order to create single pair-wise comparisons and to mitigate the unit-of-analysis error. Paired analyses were conducted for all crossover trials [31]. Where necessary, a pooled correlation coefficient was derived and used for calculation of an imputed SD for the between-treatment difference for some crossover trials. Correlation coefficients between baseline and end-of-treatment values within each individual crossover trial were derived from the reported within and between treatment SD according to a published formula [31]. These correlation coefficients were transformed into z-scores ± SD, meta-analyzed using inverse-variance weighing, and back transformed to derive the pooled correlation coefficient. Where we could not derive a calculated pooled correlation coefficient for imputing missing SDs we assumed a correlation coefficient of 0.5, as it is a conservative estimate for an expected range of 0–1. A correlation coefficient of 0.5 was assumed in the primary analysis for HbA1c due to insufficient data and in the primary analyses for fasting glucose and insulin owing to considerable heterogeneity between the derived correlation coefficients (only 2 available correlation coefficients available for pooling in both analyses). The values derived from each trial were pooled and analyzed for each endpoint (HbA1c, fasting glucose, fasting insulin, and HOMA-IR) using the generic inverse variance method with random effects models, which was used even in the absence of statistically significant between-study heterogeneity, as they yield more conservative summary effect estimates in the presence of residual heterogeneity. Exceptions were made for the use of fixed-effects models where there were <5 included trials irrespective of heterogeneity or small trials being pooled with larger more precise trials in the absence of statistically significant between-study heterogeneity. Data were expressed as mean differences (MD) with 95% CI. A two-sided p-value <0.05 was set as the level of significance for comparisons of MD.

Inter-study heterogeneity was tested using the Cochran Q-statistic and quantified using the I^2^-statistic with a significance level set at p-value <0.10. An I2<50%, I2≥50% and I2≥75% were considered to be evidence of “moderate”, “substantial” and “considerable” heterogeneity, respectively [23]. Sources of heterogeneity were explored using sensitivity and subgroup analyses. To determine whether a single trial exerted an undue influence on the overall results, sensitivity analyses were performed in which each individual trial was removed from the meta-analysis and the effect size recalculated with the remaining trials. Sensitivity analyses were also undertaken using correlation coefficients of 0.25, 0.50 and 0.75 to determine whether the overall results were robust to the use of different derived correlation coefficients in paired analyses of crossover trials. A priori subgroup analyses (continuous and categorical) were conducted for baseline values of HbA1c, fasting glucose, fasting insulin and HOMA-IR within the intervention arm, nut type, absolute fiber and saturated fat intake within the intervention arm, difference in fiber and saturated fat intake between the intervention and control arm, change in fiber and saturated fat intake from baseline within the intervention arm, dose, design, follow-up, and study quality (MQS). Post-hoc subgroup analyses were conducted for the difference in percent carbohydrate intake between the control and intervention arm (carbohydrate displacement), sex and BMI. Meta-regression was performed to assess the significance of the subgroup effects with STATA software, version 12.0 (StataCorp, College Station, TX) with a significance level set at p-value <0.05.

Publication bias was investigated by visual inspection of funnel plots and quantitatively assessed using Egger’s and Begg’s tests, where a p-value <0.05 was considered evidence of small study effects.

## Results

### Search Results

**Figure 1:**
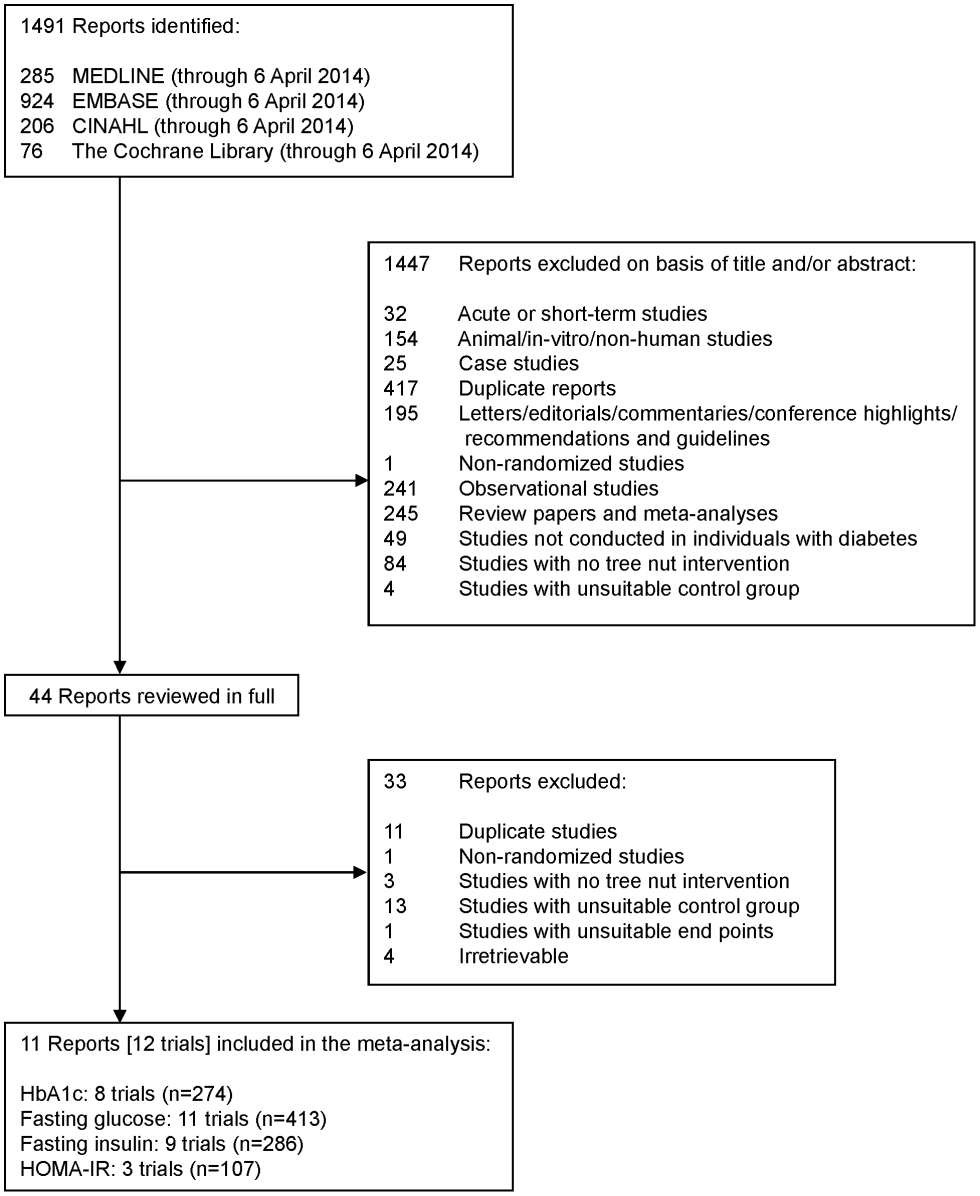
Flow of the literature. Summary of search and selection process consists of the number of studies initially identified through database and manual search, excluded based on title and abstract, reviewed in full, excluded after full review, and final number of trials included in the meta-analysis.

**Figure 1** shows the flow of the literature. The search identified a total of 1491 reports, 1447 of which were determined to be irrelevant based on review of titles and abstracts. The remaining 44 reports were retrieved and reviewed in full, of which 33 were excluded. A total of 11 reports containing 12 trials in 450 participants with diabetes [27]–[30], [32]–[38] were selected for analyses. Eight trials reported data for HbA1c (n = 274), 11 for fasting glucose (n = 413), 9 for fasting insulin (n = 286), and 3 for HOMA-IR (n = 107).

### Trial Characteristics

**Table 1** shows the characteristics of the 12 included trials (n = 450). Trials were mainly conducted in outpatient settings across 5 countries: United States (6 trials), Australia (2 trials), Iran (2 trials), and 1 trial each from Canada and Taiwan. All trials were randomized and more than half (58%) used a parallel design. Participants tended to be middle aged (median age: 57 years [range: 51–66 years]) with approximately an equal number of men and women (ratio of women to men: 1.2). Median baseline HbA1c, fasting glucose, fasting insulin, and HOMA-IR were 7.0% (53 mmol/mol), 8.1 mmol/L, 108.3 pmol/L, and 5.7, respectively. All trials were conducted in individuals with type 2 diabetes, however, in one of the trials [28] it was not clear whether all participants had diabetes. Mean diabetes duration varied from at least 1 year [30], [32], [35], [37] to ∼7–8 years [33], [34]; otherwise, it was undeclared [27]–[29], [36], [38]. The majority of trials did not explicitly provide information on how diabetes was defined stating only that diabetes had to be previously diagnosed by a physician, and/or treated for at least 1 year. Participants tended to be on antihyperglycemic medications [7 trials [27], [30], [33]–[35], [38]] but not insulin [9 trials reported insulin therapy as part of their exclusion criteria [27], [30], [32], [34]–[38]]. Four trials stated participants were to keep their medication use consistent throughout the trial [30], [33], [34], [38]; otherwise, it was not explicitly stated.

**Table 1:** Trial Characteristics,

Study, Year (Reference)		Participants*	Mean Age, y (SD)	Mean Body Weight or BMI (SD) †	Setting ‡	Design	Feeding Control §
**Lovejoy et al, 2002-HF** [27]		30 T2D (13 M, 17 W)	53.8 (10.4)	33.0 (5.5) kg/m^2^	OP, USA	C	Met
**Lovejoy et al, 2002-LF** [27]		30 T2D (13 M, 17 W)	53.8 (10.4)	33.0 (5.5) kg/m^2^	OP, USA	C	Met
**Wien et al, 2003** [28]		65 O (28 M, 37 W)			OP, USA	P	Supp
Almond			53 (2)	113 (5) kg			
Control			57 (2)	114 (5) kg			
**Tapsell et al, 2004** [37]		37 T2D (21 M, 16 W)			OP, AUS	P	Supp
Walnut			57.7 (9.0)	87.6 (12.8) kg			
Control			60.5 (8.2)	81.9 (11.2) kg			
**Tapsell et al, 2009** [36]		35 T2D (−)	54 (8.7)		OP, AUS	P	Supp
Walnut				94.3 (18.1) kg			
Control				93.9 (14.7) kg			
**Ma et al, 2010** [35]		22 T2D (−)	58.1 (9.2)	89.0 (15.5) kg	OP, USA	C	Supp
Walnut							
Control							
**Cohen et al, 2011** [30]		13 T2D (7 M, 6 W)			OP, USA	P	Supp
Almond			66 (8.1)	96.1 (21.8) kg			
Control			66 (8.7)	105.1 (29.6) kg			
**Jenkins et al, 2011** [33]		79 T2D (52 M, 27 W)			OP, CAN	P	Supp
Mixed nuts			63 (9)	80 (15) kg			
Control			61 (10)	83 (15) kg			
**Li et al, 2011** [34]		20 T2D (9 M, 11 W)	58 (8.94)	26.0 (3.13) kg/m^2^	OP, TWN	C	Met
Almond							
Control							
**Darvish Damavandi et al, 2012** [32]		43 T2D (9 M, 34 W)			OP, IRN	P	Supp
Cashew			51 (7.9)	72.1 (13.1) kg			
Control			56 (5.7)	71.9 (9.7) kg			
**Darvish Damavandi et al, 2013** [38]		48 T2D (15 M, 33 W)	55.7 (7.74)		OP, IRN	P	Supp
Hazelnut				72.13 (10.27) kg			
Control				71.98 (9.58) kg			
**Sauder et al, 2013** [29] ††† **Pistachio Control**		28 T2D (−)	56.1 (7.67)	31.2 (6.02) kg/m^2^	−, USA	C	Met
**Nut Dose, g/d (%E) ||**	**Nut Type¶**	Comparator**	**Diet††**	**Energy Balance**	**Follow-Up**	**MQS ‡‡**	**Funding Sources §§**
57–113 (**∼**18.8)	Almond	High fat diet	48∶15∶37	Neutral	4 wk	5	Agency
57–113 (**∼**18.8)	Almond	Low fat diet	60∶15∶25	Neutral	4 wk	5	Agency
84 (**∼**47.7)	Almond	Self-selected complex CHO’s		Negative	24 wk	8	Agency
			32∶29∶39 53∶29∶18				
30 (**∼**9.8)	Walnut	Low fat/modified fat diet		Neutral	6 month	6	Agency
			44∶22∶32 41∶23∶33				
30 (**∼**9.8)	Walnut	Low fat diet		Neutral	12 month	7	Agency
			41∶21∶34 42∶24∶29				
56 (**∼**20.7)	Walnut	Ad libitum diet		Neutral	8 wk	5	N/A
			39∶17∶45 43∶19∶38				
28 (**∼**17.8) **||||**	Almond	Cheese sticks	N/A	Neutral	12 wk	7	Agency
							
50–100 (**∼**25)	Mixed nuts ¶¶	NCEP Step 2 diet+Muffin		Neutral***	12 wk	8	Agency
			41∶18∶41 46∶19∶35				
56 (20)	Almond	NCEP Step 2 diet		Neutral	4 wk	5	Agency
			47∶17∶37 57∶17∶27				
30 (10)	Cashew	Regular diet		Neutral	8 wk	3	N/A
			53∶16∶33 57∶16∶27				
29 (10)	Hazelnut	Regular diet		Neutral	8 wk	4	N/A
			55∶16∶31 60∶17∶25				
**∼**71 (20)	Pistachio	Low fat diet		–	4 wk	–	–
			51∶17∶33 55∶18∶27				

BMI = body mass index; C = crossover; CHO = carbohydrate; E = energy; HF = high fat; HOMA-IR = homeostasis model assessment of insulin resistance; IP = inpatient; LF = low fat; M = men; Met = metabolic feeding control; MQS = Heyland Methodological Quality Score; N/A = not available; NCEP = National Cholesterol Education Program; O = obese and overweight; OP = outpatient; P = parallel; SD = standard deviation; Supp = supplement feeding control; T2D = type 2 diabetes; W = women; wk = week; y = years.

*The number of participants listed for each trial in this column is the number of participants that completed the trial and therefore the number used in our analyses. The baseline characteristics reported by these trials were based on the number of participants listed here with the exception of 3 trials, Tapsell et al. [36], Ma et al. [35], and Darvish Damavandi et al. [38] where the values for mean age and/or mean body weight or BMI were derived from the number of participants present at baseline, a number that was different from the number of participants that completed the trial due to a per-protocol with drop-outs analysis. The number of participants present at baseline for these trials are as follows: Tapsell et al. [36], n = 50; Ma et al. [35], n = 24; Darvish Damavandi et al. [38], n = 50; Sauder et al. [29], n = 30.

† Baseline body weight or weight (kg) while receiving the control treatment in cross over trials, and baseline body weight in each treatment group in parallel trials. Baseline BMI values (kg/m^2^) are only reported when no data on weight were available.

‡ Countries are abbreviated using three letter country codes (ISO 3166-1 alpha-3 codes).

§ Metabolic feeding control (Met) was the provision of all meals, snacks, and study supplements (tree nuts) consumed during the study under controlled conditions. Supplement feeding control (Supp) was the provision of study supplements only.

|| Doses and % E (energy) preceded by “∼” represent values calculated on the basis of average reported energy intake of participants and average reported energy values of tree nuts from the USDA National Nutrient Database [59].

¶ All nut types were provided in whole form with the exception of 2 trials: Lovejoy et al. [27] and Li et al. [34], which incorporated tree nuts into various entrées and snack foods (i.e. muffins, trail mixes, deserts, etc.).

**Comparators refers to 1) reference food(s) energy matched in exchange for tree nuts or 2) isocaloric control diet similar to the intervention diet but without tree nuts.

†† Planned energy from Carbohydrate:Protein:Fat. Measured energy end values from carbohydrate, protein, and fat are reported only if the study did not state the planned energy of prescribed diets.

‡‡ Trials with a MQS score ≥8 were considered to be of higher quality.

§§ Agency funding is that from government, university, or not-for-profit health agency sources. None of the trialists declared any conflicts of interest with the exception of Jenkins et al. [33] and Darvish Damavandi et al. [32].

|||| In this study participants randomized into the almond group were instructed to consume this dose 5 days/week.

¶¶ Mixed nuts included almonds, cashews, hazelnuts, macadamia nuts, peanuts, pecans, pistachios, walnuts.

***43% of the participants were obese and wished to lose weight; although this was not a weight loss study, they were given advice on portion size and fat intake to help them meet their weight-reduction objective.

††† Data for this study was limited since the study’s conferences abstract and correspondence with the authors were the only sources of available data.

Laboratory measurements of glycemic endpoints across trials varied. HbA1c was measured by high-performance liquid chromatography (HPLC) in 1 trial [33], immunoassay in 3 trials [27], [30], “standard procedures” in 1 trial [35], or unspecified methods in the remaining 3 trials [29], [36], [37]. Fasting glucose was measured by enzymatic methods in 8 trials [27], [28], [30], [32]–[34], [38], “standard procedures” in 1 trial [35], or unspecified methods in the remaining 2 trials [29], [36]. Fasting insulin was measured by a radioimmunoassay in 2 trials [28], [30], an immunoassay in 4 trials [27], [32], [34], “standard procedures” in 1 trial [35], or unspecified methods in the remaining 2 trials [29], [36]. HOMA-IR was calculated according to the standard formula (insulin × glucose/22.5) in 3 trials [28], [34], [35].

Tree nut type varied among the trials: 5 trials (42%) included an intervention with almonds, 1 trial each with cashews, hazelnuts, pistachios, and mixed nuts (including almonds, cashews, hazelnuts, macadamia nuts, peanuts, pecans, pistachios, walnuts), and 3 trials (25%) with walnuts. Tree nuts were consumed as whole nuts in majority of the trials with the exception of 2 trials [27], [34] where tree nuts were provided in meal form as part of entrées and snack foods (i.e. muffins, trail mix, deserts, etc.). The median dose was ∼56 g/d (range: 28–85 g/d). The method of increasing tree nuts while maintaining isocaloric comparisons between arms differed across trial protocols: 3 trials replaced or emphasized reduction in carbohydrate foods [28], [29], [33], 1 replaced sources of dairy (cheese) [30], 1 exchanged tree nuts for protein-rich foods and oils/spreads [37], 1 reduced portions of meats and amount of visible fats (i.e. oils, margarines, and butter) [38], and 6 either did not specify, did not provide specific instructions on food replacement, or information was unavailable [27], [32], [34]–[36] The background diets consisted of 32–60% energy (E) from carbohydrate, 15–29% E protein, and 18–45% E fat with a median fiber and saturated fat intake of 24.6 g/d (range: 11.4–32 g/d) and 7.4% E (range: 3–12.5%E), respectively, in the comparator diets, and 27.6 g/d (range: 16.5–35.6g/d) and 6.9% E (range: 3–10.9%E), respectively, in the tree nut enriched diets. One trial was a weight reduction intervention [28] and 1 trial provided the option of weight reduction during the study period in those who wished to lose weight [33]. In terms of feeding control, 4 trials (33%) were metabolically controlled (i.e. all foods were provided) and 8 trials (67%) provided test food supplements. The median follow-up duration was 8 weeks (range: 4–48 weeks).

The majority of trials (75%) were considered to be of poor quality (MQS<8). Absence of double-blinding and high dropout rates contributed to lower scores (**Table S2**). Trials were judged as having a ‘low’ or ‘unclear risk bias’ for majority of the domains measured by the Cochrane Risk of Bias Tool. A few trials were considered ‘high risk of bias’ due to incomplete outcome data (**Figure S1**). Majority of the trials were funded by agency alone (73%); 3 trials did not declare their source of funding [32], [35], [38] and for 1 trial information was unavailable [29].

### Hemoglobin A1c (HbA1c)

**Figure 2:**
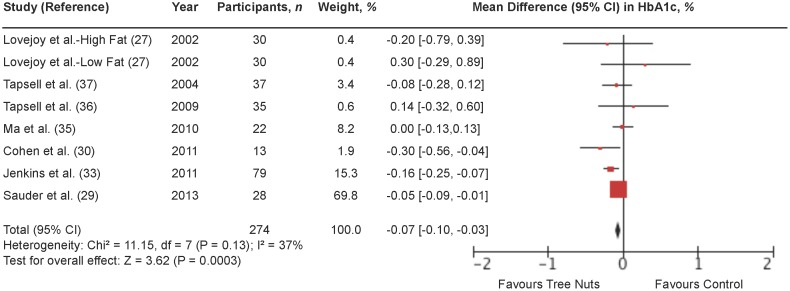
Forest plot of randomized controlled trials investigating the effect of diets supplemented with tree nuts on HbA1c in individuals with type 2 diabetes. Pooled effect estimate (*diamond*) for HbA1c (%). Data are expressed as weighted mean differences (MD) with 95% CIs, using the generic inverse-variance fixed effects model. Paired analyses were applied to all crossover trials. Inter-study heterogeneity was tested by the Cochran Q-statistic and quantified by I^2^ at a significance level of P<0.10. n = number of participants in each treatment group.

**Figure 2** shows a forest plot of the pooled effect of tree nuts on HbA1c in individuals with type 2 diabetes. Diets emphasizing tree nuts significantly lowered HbA1c in comparison to control diets (MD = −0.07% [95% CI: −0.10, −0.03%]; P = 0.0003) with no significant evidence of inter-study heterogeneity (I^2^ = 37%; P = 0.13). Systematic removal of individual trials did not alter the results. Sensitivity analyses using different correlation coefficients in paired analyses of crossover trials (0.25 and 0.75) did not alter the significance of the pooled effect size.

**Table S3 and Figure S2** shows the results of continuous and categorical subgroup analyses for the effect of tree nuts on HbA1c. Meta-regression analyses did not reveal any statistically significant subgroup effects.

### Fasting glucose

**Figure 3:**
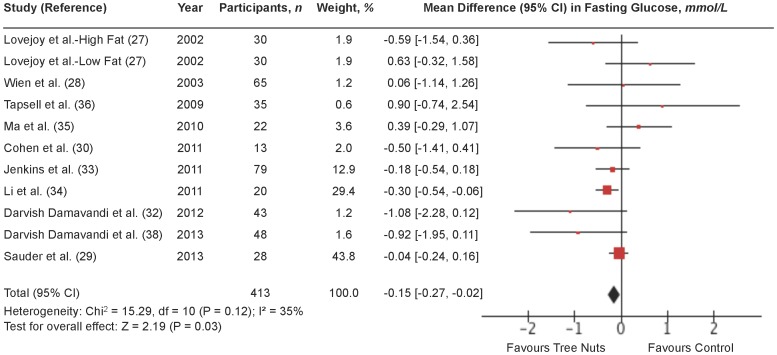
Forest plot of randomized controlled trials investigating the effect of diets supplemented with tree nuts on fasting glucose in individuals with type 2 diabetes. Pooled effect estimate (*diamond*) for fasting glucose (mmol/L). Data are expressed as weighted mean differences (MD) with 95% CIs, using the generic inverse-variance fixed effects model. Paired analyses were applied to all crossover trials. Inter-study heterogeneity was tested by the Cochran Q-statistic and quantified by I^2^ at a significance level of P<0.10. n = number of participants in each treatment group.

**Figure 3** shows a forest plot of the pooled effect of tree nuts on fasting glucose in individuals with type 2 diabetes. Diets emphasizing tree nuts significantly lowered fasting glucose in comparison to control diets (MD = −0.15 mmol/L [95% CI: −0.27, −0.02 mmol/L]; P = 0.03) with no significant evidence of inter-study heterogeneity (I^2^ = 35%; P = 0.12). Sensitivity analyses showed that individual removal of any of the following 3 trials changed the pooled effect size from significant to non-significant: Jenkins et al. [33] (MD = −0.14 mmol/L [95% CI: −0.28, −0.00 mmol/L]; P = 0.05] with moderate inter-study heterogeneity (I^2^ = 41%; P = 0.08); Li et al. [34] (MD = −0.08 mmol/L [95% CI: −0.23, 0.07 mmol/L]; P = 0.31) with no significant evidence of inter-study heterogeneity (I^2^ = 31%; P = 0.16) and Darvish Damavandi et al. [38] (MD = −0.13 mmol/L [95% CI: −0.26, −0.00 mmol/L]; P = 0.05) with no significant evidence of inter-study heterogeneity (I^2^ = 31%; P = 0.16). Sensitivity analyses using different correlation coefficients in paired analyses of crossover trials showed that a correlation coefficient of 0.25 did not alter the significance of the pooled effect size, but a correlation coefficient of 0.75 changed the pooled effect size from significant to non-significant (MD = −0.14 mmol/L [95% CI: −0.36, 0.08 mmol/L]; P = 0.20) and resulted in moderate inter-study heterogeneity (I^2^ = 48%; P = 0.04).

**Table S4 and Figure S3** shows the results of continuous and categorical subgroup analyses for the effect of tree nuts on fasting glucose. Meta-regression analyses did not reveal any statistically significant subgroup effects.

### Fasting insulin

**Figure 4:**
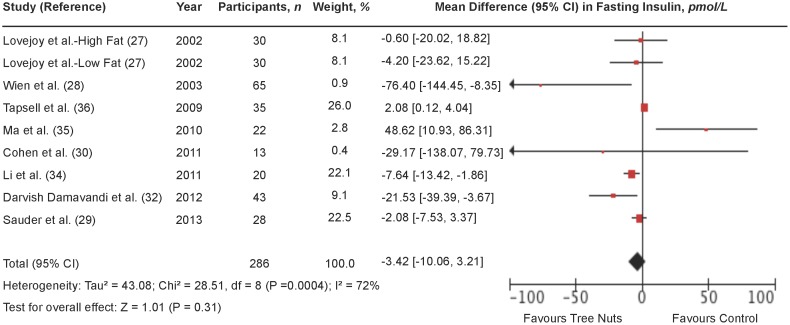
Forest plot of randomized controlled trials investigating the effect of diets supplemented with tree nuts on fasting insulin in individuals with type 2 diabetes. Pooled effect estimate (*diamond*) for fasting insulin (pmol/L). Data are expressed as weighted mean differences (MD) with 95% CIs, using the generic inverse-variance random-effects model. Paired analyses were applied to all crossover trials. Inter-study heterogeneity was tested by the Cochran Q-statistic and quantified by I^2^ at a significance level of P<0.10. n = number of participants in each treatment group.

**Figure 4** shows a forest plot of the pooled effect of tree nuts on fasting insulin in individuals with type 2 diabetes. Diets emphasizing tree nuts had no significant overall effect on fasting insulin in comparison to control diets (MD = −3.42 pmol/L [95% CI: −10.06, 3.21 pmol/L]; P = 0.31) with substantial evidence of inter-study heterogeneity (I^2^ = 72%; P = 0.0004). Systematic removal of individual trials did not alter the results. Sensitivity analyses using different correlation coefficients in paired analyses of crossover trials (0.25 and 0.75) did not alter the significance of the pooled effect size.

**Table S5 and Figure S4** shows the results of continuous and categorical subgroup analyses for the effect of tree nuts on fasting insulin. Meta-regression analyses did not reveal any statistically significant subgroup effects.

### Homeostasis model assessment of insulin resistance (HOMA-IR)

**Figure 5:**
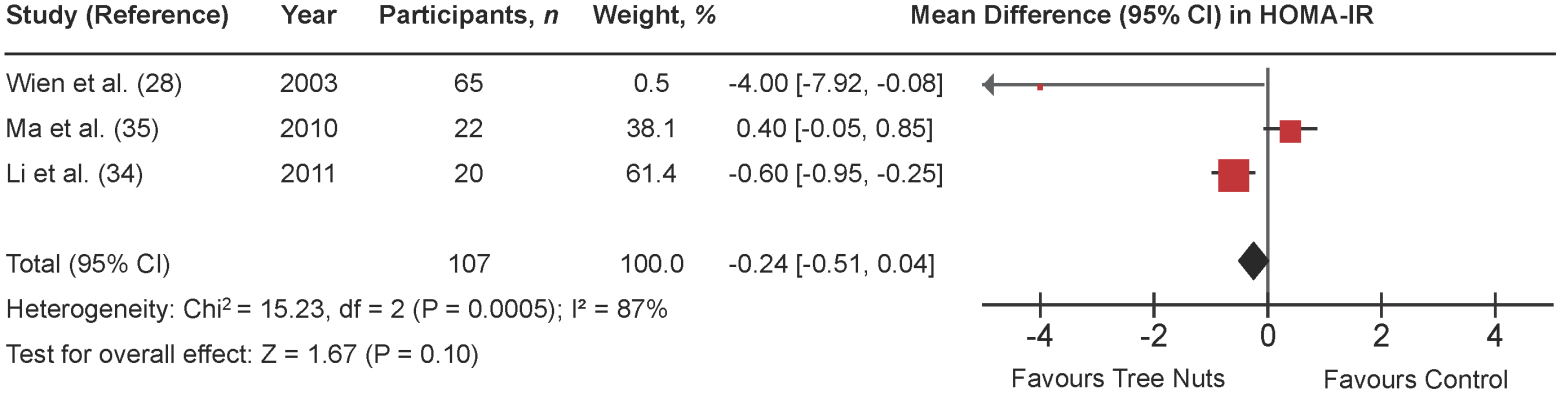
Forest plots of randomized controlled trials investigating the effect of diets supplemented with tree nuts on HOMA-IR in individuals with type 2 diabetes. Pooled effect estimate (*diamond*) for homeostasis model assessment of insulin resistance (HOMA-IR). Data are expressed as weighted mean differences (MD) with 95% CIs, using the generic inverse-variance fixed-effects model. Paired analyses were applied to all crossover trials. Inter-study heterogeneity was tested by the Cochran Q-statistic and quantified by I^2^ at a significance level of P<0.10. n = number of participants in each treatment group.

**Figure 5** shows a forest plot of the pooled effect of tree nuts on HOMA-IR in individuals with type 2 diabetes. Diets emphasizing tree nuts had no significant effect on HOMA-IR in comparison to control diets (MD = −0.24 [95% CI: −0.51, 0.04]; P = 0.10) with considerable evidence of inter-study heterogeneity (I^2^ = 87%; P = 0.0005). Sensitivity analyses showed that removal of the trial Ma et al. (27) changed the pooled effect size from non-significant to significant (MD = −0.63 [95% CI: −0.98, −0.27]; P = 0.0005) with substantial evidence of inter-study heterogeneity (I^2^ = 65%; P = 0.09).

**Table S6 and Figure S5** shows the results of continuous and categorical subgroup analyses for the effect of tree nuts on HOMA-IR. Meta-regression analyses did not reveal any statistically significant subgroup effects.

### Publication bias

**Figure 6:**
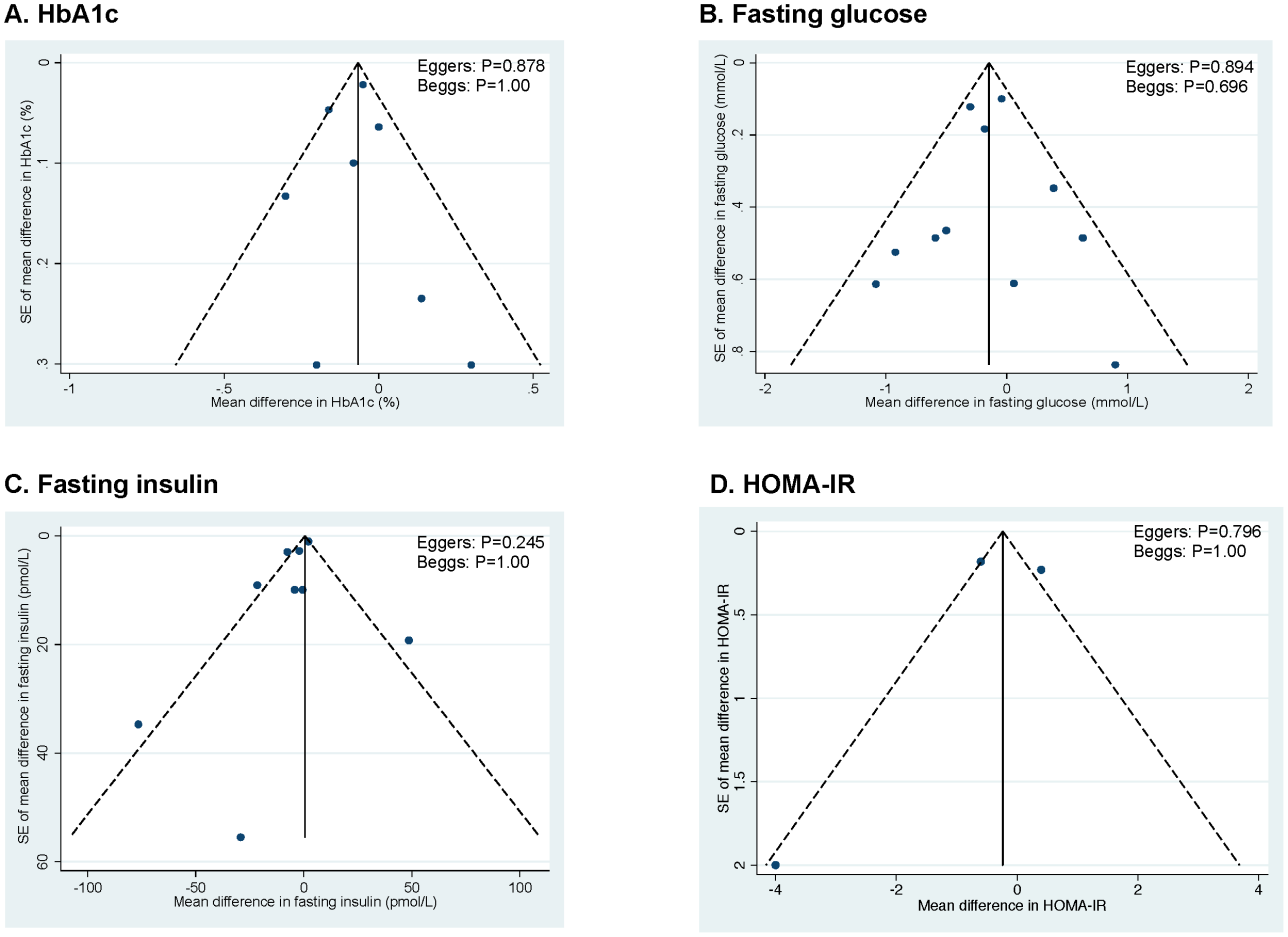
Publication bias funnel plots. Publication bias funnel plots for HbA1c (A), fasting glucose (B), fasting insulin (C), and HOMA-IR (D). The solid line represents the pooled effect estimate expressed as the weighted mean difference for each analysis. The dashed lines represent pseudo-95% confidence limits. P-values displayed in the top right corner of each funnel plot are derived from quantitative assessment of publication bias by Egger’s and Begg’s tests.

**Figure 6** **(A–D)** shows the funnel plots for each glycemic endpoint. Visual inspection of funnel plots revealed asymmetry for fasting insulin, suggesting study effects favouring the tree nut intervention. Egger’s and Begg’s tests did not reveal significant evidence of publication bias for any of the primary analyses. With one exception, these tests should be interpreted with caution, as they were based on <10 trials.

## Discussion

To our knowledge this is the first systematic review and meta-analysis of randomized controlled trials to assess the effect of tree nut consumption on HbA1c, fasting glucose, fasting insulin, and/or HOMA-IR in individuals with type 2 diabetes. We included 12 randomized controlled dietary trials looking at the effect of tree nuts on these 4 endpoints in 450 predominantly middle-aged adults. Pooled analyses showed an overall significant lowering of HbA1c of −0.07% and fasting glucose of −0.15 mmol/L at a median dose of 56 g/d over a median duration of ∼8 weeks. Although significant advantages for fasting insulin and HOMA-IR were not seen, the direction of effect favoured the tree nut intervention.

There is limited existing research looking at the ability of nuts to improve glycemic control over the long term. Previous studies looking at dietary patterns including nuts, such as the Mediterranean and the Dietary Approaches to Stop Hypertension (DASH) dietary pattern, are consistent with our findings. In a systematic review of 5 randomized controlled trials [39], as well as several individual randomized controlled trials [40]–[43] including people with type 2 diabetes, a Mediterranean dietary pattern emphasizing nuts showed decreases in HbA1c (from −0.1% to −0.6% absolute reduction), fasting glucose, and the need for antihyperglycemic drugs over a 4 year period [40], [44] in comparison to a conventional dietary pattern. Improvements in other markers related to glycemic control, such as the adiponectin/leptin ratio, have also been reported [45]. Similar findings were found regarding the DASH dietary pattern emphasizing nuts, where a randomized controlled trial conducted in people with type 2 diabetes showed that compared with a control diet (matched for a moderate sodium intake), the DASH dietary pattern was able to improve HbA1c (an absolute reduction of −1.2%) and fasting glucose (−0.92 mmol/L) over an 8 week period [46].

The ability of tree nuts to improve glycemic control may relate to a carbohydrate displacement mechanism by which tree nuts reduce the glycemic load of the diet by displacing high glycemic-index carbohydrates. Of the 3 trials that showed a significant lowering in HbA1c, the two trials contributing the greatest amount of weight to the analysis (>80% collectively) [29], [33] investigated the effect of tree nuts as a means of displacing carbohydrate by ≥5% of energy [33]. The addition of similar trials in future meta-analyses would be expected to strengthen our results, however, it is not clear whether this lowering would reach a clinically meaningful threshold of ≥0.3% [10]. Other proposed factors relate to the micro- and macronutrient profile of nuts, such as magnesium and monounsaturated fat (MUFA) content. Magnesium content of tree nuts can range from 121–376 mg and MUFA from 9–59 g per 100 g [1], providing approximately between 30% to 94% and 14% and 91% of the Daily Value (DV) for magnesium and total fat, respectively [47]. Meta-analyses of prospective cohort studies and randomized double-blind controlled trials looking at magnesium intake in individuals with type 2 diabetes support decreases in diabetes risk [48], [49], as well as benefits for glycemic control [50]. Magnesium is thought to play a key role in insulin-mediated glucose uptake [51], [52], where animal studies have shown poor intracellular magnesium concentrations to result in defective tyrosine-kinase activity at the insulin-receptor level and therefore impairing insulin action [52], [53]. Similarly, in a meta-analysis of randomized controlled trials looking at the effects of MUFA on glycemic control in individuals with abnormal glucose metabolism, diets high in MUFA were shown to significantly reduce HbA1c [54]. Human trials and animal studies suggest that MUFA may also be involved in the insulin-signaling pathway by playing a role in membrane translocation of glucose transporters in skeletal muscle, as well as by buffering β-cell hyperactivity and insulin resistance [54]–[56]. Although our results do not show significant improvements in insulin resistance by HOMA or fasting insulin levels, there were a limited number of trials and a significant amount of heterogeneity present in the primary analyses. In addition, neither endpoint is a good marker of peripheral insulin sensitivity [57]. Overall, these proposed mechanisms suggest that carbohydrate displacement, magnesium and MUFA content of nuts may be contributing factors in facilitating the effect seen on glycemic control.

Heterogeneity was not explained by majority of the subgroup analyses, where only two subgroups, follow-up and study design in the categorical subgroup analyses for HbA1c, approached significance. Trials of ≥12 weeks showed a greater lowering-effect of tree nuts on HbA1c than trials of <12 weeks. These results suggest that tree nut consumption over a longer period (from 12 weeks to 24 months among available trials) may lead to greater improvements in glycemic control. Similarly, trials using a parallel design showed a greater lowering-effect of tree nuts on HbA1c than those using a crossover design. The smaller effect of tree nuts in crossover designs may be attributed to carry-over effects [23]. In our meta-analysis, all 5 trials with a crossover design contained a washout period ranging from 1–8 weeks. Since HbA1c reflects mean glycemia for the previous 3 months [58], it is not certain whether this is long enough to diminish any potential carry-over effects.

Several limitations exist in the present meta-analysis that complicates the interpretation of our results. First, it is uncertain whether the length of the follow-up period in these trials is enough time for tree nuts to significantly alter glycemic control. HbA1c levels reflect blood glucose levels in the preceding 3 months (∼90 days or 12 weeks) [58], whereas more than half of the trials (54%) were less than 12 weeks in duration. Second, there was evidence of substantial and considerable between study heterogeneity in the overall primary analyses for fasting insulin and HOMA-IR, respectively, which was not explained by any of the *a priori* and post-hoc subgroup analyses. In addition, majority of subgroup analyses were underpowered and it was not possible to assess the effect of other factors that may influence glycemic control (i.e. growing conditions of tree nuts) due to unavailability of data. Third, the majority of the trials (81%) were of poor study quality (MQS<8), however, no effect modification by study quality was found in the subgroup analyses. Fourth, a portion of the trials (27%) did not focus on glycemic control endpoints as their primary outcome.

In conclusion, the present systematic review and meta-analysis of randomized controlled trials shows that a daily median intake of 56 g (∼2 ounces or ∼½ cup) of tree nuts over a median duration of 8 weeks significantly reduces HbA1c and fasting glucose in individuals with type 2 diabetes. Although significant advantages were not seen for fasting insulin and HOMA-IR, the direction of effect favoured tree nuts. The greatest advantages appear to be seen in trials using tree nuts to displace high-glycemic index carbohydrate to affect a low-glycemic load diet. To address the sources of uncertainty in our analyses, there is a need for large, longer, higher quality trials using tree nuts to lower the glycemic load of the diet by displacing high-glycemic index carbohydrates with a specific focus on glycemic endpoints as a primary outcome. The inclusion of such trials in future meta-analyses will help guide the development of nutrition recommendations and health claims, as well as the planning of future trials. Overall, our data support the inclusion of tree nuts as part of a healthy diet for the management of glycemia in individuals with type 2 diabetes.

## Supporting Information

Figure S1 **Cochrane Risk of Bias Graph.** Risk of bias graph: review authors’ judgments about each risk of bias item presented as percentages across all included studies (with the exception of Sauder et al. [29]).
(TIFF)

Figure S2 **Categorical** ***a priori*** **and post-hoc subgroup analyses for HbA1c.** CHO = carbohydrate; N = number of subjects; MQS = Heyland Methodological Quality Score; SFA = saturated fatty acid. Point estimates for each subgroup level (diamonds) are the pooled effect estimates. The dashed line represents the pooled estimate for the overall (total) analysis. The residual I^2^ value indicates heterogeneity unexplained by the subgroup. Pairwise between-subgroup mean differences (95%CIs) for nut type were as follows: 0.15 [−0.20, 0.49] (1 vs. 4); 0.18 [−0.18, 0.55] (1 vs. 5); 0.04 [−0.33, 0.40] (1 vs. 6); −0.03 [−0.23, 0.16] (4 vs. 5); 0.11 [−0.09, 0.31] (4 vs. 6); 0.14 [−0.09, 0.37] (5 vs. 6). Absolute intakes represent intakes within the treatment arm. Between arm differences represent the difference between the treatment (T) and control (C) arm (T–C). Within arm differences represent the difference between end (E) and baseline (B) values within the treatment arm (E–B). *Statistically significant between subgroups (P<0.05).
(PDF)

Figure S3 **Categorical** ***a priori*** **and post-hoc subgroup analyses for fasting glucose.** CHO = carbohydrate; N = number of subjects; MQS = Heyland Methodological Quality Score; SFA = saturated fatty acid. Point estimates for each subgroup level (diamonds) are the pooled effect estimates. The dashed line represents the pooled estimate for the overall (total) analysis. The residual I^2^ value indicates heterogeneity unexplained by the subgroup. Pairwise between-subgroup mean differences (95%CIs) for nut type were as follows: −0.81 [−2.41, 0.79] (1 vs. 2); −0.65 [−2.03, 0.73] (1 vs. 3); 0.23 [−0.15, 0.61] (1 vs. 4); 0.73 [−0.14, 1.60] (1 vs. 5); 0.09 [−0.46, 0.64] (1 vs. 6); −0.16 [−2.23, 1.91] (2 vs. 3); −1.04 [−2.64, 0.56] (2 vs. 4); −1.54 [−3.32, 0.23] (2 vs. 5); −0.90 [−2.55, 0.75] (2 vs. 6); −0.88 [−2.25, 0.49] (3 vs. 4); −1.38 [−2.97, 0.20] (3 vs. 5); −0.74 [−2.17, 0.69] (3 vs. 6); −0.50 [−1.37, 0.36] (4 vs. 5); 0.14 [−0.40, 0.68] (4 vs. 6); 0.64 [−0.31, 1.59] (5 vs. 6). Absolute intakes represent intakes within the treatment arm. Between arm differences represent the difference between the treatment (T) and control (C) arm (T–C). Within arm differences represent the difference between end (E) and baseline (B) values within the treatment arm (E–B). *Statistically significant between subgroups (P<0.05).
(PDF)

Figure S4 **Categorical a priori and post-hoc subgroup analyses for fasting insulin.** CHO = carbohydrate; N = number of subjects; MQS = Heyland Methodological Quality Score; SFA = saturated fatty acid. Point estimates for each subgroup level (diamonds) are the pooled effect estimates. The dashed line represents the pooled estimate for the overall (total) analysis. The residual I^2^ value indicates heterogeneity unexplained by the subgroup. Pairwise between-subgroup mean differences (95%CIs) for nut type were as follows: −13.00 [−65.37, 39.37] (1 vs. 2); 6.45 [−38.57, 51.47] (1 vs. 4); 20.81 [−19.98, 61.59] (1 vs. 5); −19.45 [−79.26, 40.36] (2 vs. 4); −31.81 [−90.50, 22.88] (2 vs. 5); −14.36 [−64.34, 35.63] (4 vs. 5). Absolute intakes represent intakes within the treatment arm. Between arm differences represent the difference between the treatment (T) and control (C) arm (T–C). Within arm differences represent the difference between end (E) and baseline (B) values within the treatment arm (E–B). * Statistically significant between subgroups (P<0.05).
(PDF)

Figure S5 **Categorical** ***a priori*** **and post-hoc subgroup analyses for HOMA-IR.** CHO = carbohydrate; N = number of subjects; MQS = Heyland Methodological Quality Score; SFA = saturated fatty acid. Point estimates for each subgroup level (diamonds) are the pooled effect estimates. The dashed line represents the pooled estimate for the overall (total) analysis. The residual I^2^ value indicates heterogeneity unexplained by the subgroup. Absolute intakes represent intakes within the treatment arm. Between arm differences represent the difference between the treatment (T) and control (C) arm (T–C). Within arm differences represent the difference between end (E) and baseline (B) values within the treatment arm (E–B). * Statistically significant between subgroups (P<0.05).
(PDF)

Table S1 **Search strategy.** For all databases, the original search was 23 May 2012; updated searches were performed 14 May 2013 and 6 April 2014.
(DOCX)

Table S2 Study Quality Assessment using the Heyland MQS*. HF = high fat diet; LF = low fat diet; MQS = Methodological Quality Score. * The Heyland MQS assigns a score of 0 or 1 or from 0 to 2 over 9 categories of quality related to study design, sampling procedures, and interventions for a total of 13 points. Trials that scored ≥8 were considered to be of higher quality [25]. † Randomization was scored 2 points for being randomized with the methods described, 1 point for being randomized without the methods described, or 0 points for being neither randomized nor having the methods described. Blinding was scored 1 point for being double-blind or 0 points for “other.” Analysis was scored 2 points for being intention-to-treat; all other types of analyses scored 0 points. ‡ Sample selection was scored 1 point for being consecutive eligible or 0 points for being preselected or indeterminate. Sample comparability was scored 1 point for being comparable or 0 points for not being comparable at baseline. Follow-up was scored 1 point for being 100% or 0 points for <100%. § Treatment protocol was scored 1 point for being reproducibly described or 0 points for being poorly described. Co-interventions were scored 2 points for being described and equal, 1 point for being described but unequal or indeterminate, or 0 points for not being described. Treatment crossovers (where participants were switched from the control treatment to the experimental treatment) were scored 2 points for being <10%, 1 point for being >10%, and 0 points for not being described. || Study quality for this study was not assessed since data for this study was limited (the study’s conferences abstract and correspondence with the authors were the only sources of available data).
(DOCX)

Table S3 **Continuous** ***a priori*** **and post-hoc subgroup analyses for HbA1c.** BMI = body mass index; CHO = carbohydrate; E = energy; M = males; N = number of subjects; No. = number; SFA = saturated fatty acid. β is the slope derived from subgroup analyses on meta-regression analyses and represents the treatment effect of tree nuts for each subgroup. The residual I^2^ value indicates heterogeneity unexplained by the subgroup. Absolute intakes represent intakes within the treatment arm. Between arm differences represent the difference between the treatment (T) and control (C) arm (T–C). Within arm differences represent the difference between end (E) and baseline (B) values within the treatment arm (E–B). *Statistically significant between subgroups (P<0.05).
(DOCX)

Table S4 **Continuous** ***a priori*** **and post-hoc subgroup analyses for fasting glucose.** BMI = body mass index; CHO = carbohydrate; E = energy; M = males; N = number of subjects; No. = number; SFA = saturated fatty acid. β is the slope derived from subgroup analyses on meta-regression analyses and represents the treatment effect of tree nuts for each subgroup. The residual I^2^ value indicates heterogeneity unexplained by the subgroup. Absolute intakes represent intakes within the treatment arm. Between arm differences represent the difference between the treatment (T) and control (C) arm (T–C). Within arm differences represent the difference between end (E) and baseline (B) values within the treatment arm (E–B). * Statistically significant between subgroups (P<0.05).
(DOCX)

Table S5 **Continuous a priori and post-hoc subgroup analyses for fasting insulin.** BMI = body mass index; CHO = carbohydrate; E = energy; M = males; N = number of subjects; No. = number; SFA = saturated fatty acid. β is the slope derived from subgroup analyses on meta-regression analyses and represents the treatment effect of tree nuts for each subgroup. The residual I^2^ value indicates heterogeneity unexplained by the subgroup. Absolute intakes represent intakes within the treatment arm. Between arm differences represent the difference between the treatment (T) and control (C) arm (T–C). Within arm differences represent the difference between end (E) and baseline (B) values within the treatment arm (E–B). * Statistically significant between subgroups (P<0.05).
(DOCX)

Table S6 **Continuous a priori and post-hoc subgroup analyses for HOMA-IR.** BMI = body mass index; CHO = carbohydrate; E = energy; N = number of subjects; No. = number; SFA = saturated fatty acid. β is the slope derived from subgroup analyses on meta-regression analyses and represents the treatment effect of tree nuts for each subgroup. The residual I^2^ value indicates heterogeneity unexplained by the subgroup. Absolute intakes represent intakes within the treatment arm. Between arm differences represent the difference between the treatment (T) and control (C) arm (T–C). Within arm differences represent the difference between end (E) and baseline(B) values within the treatment arm (E–B). * Statistically significant between subgroups (P<0.05).
(DOCX)

Checklist S1 **CONSORT checklist.**
(DOC)

Protocol S1 **Trial protocol.**
(PDF)
