# Correction: Effect of Tree Nuts on Glycemic Control in Diabetes: A Systematic Review and Meta-Analysis of Randomized Controlled Dietary Trials

**DOI:** 10.1371/journal.pone.0109224

**Published:** 2014-09-23

**Authors:** The PLOS ONE Staff

There are several errors in Table 1 of the published article. A corrected Table 1 can be seen here.

**Table 1:** Trial Characteristics.

Study, Year (Reference)	Participants*	Mean Age, y (SD)	Mean Body Weight or BMI (SD)†	Setting‡	Design	Feeding Control§
Lovejoy et al, 2002-HF [27]	30 T2D (13 M, 17 W)	53.8 (10.4)	33.0 (5.5) kg/m2	OP, USA	C	Met
Lovejoy et al, 2002-LF [27]	30 T2D (13 M, 17 W)	53.8 (10.4)	33.0 (5.5) kg/m2	OP, USA	C	Met
Wien et al, 2003 [28]	65 O (28 M, 37 W)			OP, USA	P	Supp
Almond		53 (2)	113 (5) kg			
Control		57 (2)	114 (5) kg			
Tapsell et al, 2004 [37]	37 T2D (21 M, 16 W)			OP, AUS	P	Supp
Walnut		57.7 (9.0)	87.6 (12.8) kg			
Control		60.5 (8.2)	81.9 (11.2) kg			
Tapsell et al, 2009 [36]	35 T2D (-)	54 (8.7)		OP, AUS	P	Supp
Walnut			94.3 (18.1) kg			
Control			93.9 (14.7) kg			
Ma et al, 2010 [35]	22 T2D (-)	58.1 (9.2)	89.0 (15.5) kg	OP, USA	C	Supp
Walnut						
Control						
Cohen et al, 2011 [30]	13 T2D (7 M, 6 W)			OP, USA	P	Supp
Almond		66 (8.1)	96.1 (21.8) kg			
Control		66 (8.7)	105.1 (29.6) kg			
Jenkins et al, 2011 [33]	79 T2D (52 M, 27 W)			OP, CAN	P	Supp
Mixed nuts		63 (9)	80 (15) kg			
Control		61 (10)	83 (15) kg			
Li et al, 2011 [34]	20 T2D (9 M, 11 W)	58 (8.94)	26.0 (3.13) kg/m2	OP, TWN	C	Met
Almond						
Control						
Darvish Damavandi et al, 2012 [32]	43 T2D (9 M, 34 W)			OP, IRN	P	Supp
Cashew		51 (7.9)	72.1 (13.1) kg			
Control		56 (5.7)	71.9 (9.7) kg			
Darvish Damavandi et al, 2013 [38]	48 T2D (15 M, 33 W)	55.7 (7.74)		OP, IRN	P	Supp
Hazelnut			72.13 (10.27) kg			
Control			71.98 (9.58) kg			
Sauder et al, 2013 [29] †††	28 T2D (-)	56.1 (7.67)	31.2 (6.02) kg/m2	-, USA	C	Met
Pistachio						
Control						
Nut Dose, g/d (%E) ||	Nut Type¶	Comparator**	Diet††	Energy Balance	Follow-Up	MQS ‡‡	Funding Sources§§
57-113 (∼18.8)	Almond	High fat diet	48:15:37	Neutral	4 wk	5	Agency
57-113 (∼18.8)	Almond	Low fat diet	60:15:25	Neutral	4 wk	5	Agency
84 (∼47.7)	Almond	Self-selected complex CHO’s		Negative	24 wk	8	Agency
			32:29:39				
			53:29:18				
30 (∼9.8)	Walnut	Low fat/modified fat diet		Neutral	6 month	6	Agency
			44:22:32				
			41:23:33				
30 (∼9.8)	Walnut	Low fat diet		Neutral	12 month	7	Agency
			41:21:34				
			42:24:29				
56 (∼20.7)	Walnut	Ad libitum diet		Neutral	8 wk	5	N/A
			39:17:45				
			43:19:38				
28 (∼17.8) ||||	Almond	Cheese sticks	N/A	Neutral	12 wk	7	Agency
50-100 (∼25)	Mixed nuts¶¶	NCEP Step 2 diet + Muffin		Neutral***	12 wk	8	Agency
			41:18:41				
			46:19:35				
56 (20)	Almond	NCEP Step 2 diet		Neutral	4 wk	5	Agency
			47:17:37				
			57:17:27				
30 (10)	Cashew	Regular diet		Neutral	8 wk	3	N/A
			53:16:33				
			57:16:27				
29 (10)	Hazelnut	Regular diet		Neutral	8 wk	4	N/A
			55:16:31				
			60:17:25				
∼71 (20)	Pistachio	Low fat diet		-	4 wk	-	-
			51:17:33				
			55:18:27				

BMI  =  body mass index; C  =  crossover; CHO  =  carbohydrate; E  =  energy; HF  =  high fat; HOMA-IR  =  homeostasis model assessment of insulin resistance; IP  =  inpatient; LF  =  low fat; M  =  men; Met  =  metabolic feeding control; MQS  =  Heyland Methodological Quality Score; N/A  =  not available; NCEP  =  National Cholesterol Education Program; O  =  obese and overweight; OP  =  outpatient; P  =  parallel; SD  =  standard deviation; Supp  =  supplement feeding control; T2D  =  type 2 diabetes; W  =  women; wk  =  week; y = years

* The number of participants listed for each trial in this column is the number of participants that completed the trial and therefore the number used in our analyses. The baseline characteristics reported by these trials were based on the number of participants listed here with the exception of 3 trials, Tapsell et al.[36], Ma et al.[35], and Darvish Damavandi et al.[38] where the values for mean age and/or mean body weight or BMI were derived from the number of participants present at baseline, a number that was different from the number of participants that completed the trial due to a per-protocol with drop-outs analysis. The number of participants present at baseline for these trials are as follows: Tapsell et al.[36], n = 50; Ma et al.[35], n = 24; Darvish Damavandi et al.[38], n = 50; Sauder et al.[29], n = 30.

† Baseline body weight or weight (kg) while receiving the control treatment in cross over trials, and baseline body weight in each treatment group in parallel trials. Baseline BMI values (kg/m^2^) are only reported when no data on weight were available.

‡ Countries are abbreviated using three letter country codes (ISO 3166-1 alpha-3 codes).

§ Metabolic feeding control (Met) was the provision of all meals, snacks, and study supplements (tree nuts) consumed during the study under controlled conditions. Supplement feeding control (Supp) was the provision of study supplements only.

|| Doses and % E (energy) preceded by " ∼ " represent values calculated on the basis of average reported energy intake of participants and average reported energy values of tree nuts from the USDA National Nutrient Database [59].

¶ All nut types were provided in whole form with the exception of 2 trials: Lovejoy et al. [27] and Li et al. [34], which incorporated tree nuts into various entrées and snack foods (i.e. muffins, trail mixes, deserts, etc.).

** Comparators refers to 1) reference food(s) energy matched in exchange for tree nuts or 2) isocaloric control diet similar to the intervention diet but without tree nuts.

†† Planned energy from Carbohydrate:Protein:Fat. Measured energy end values from carbohydrate, protein, and fat are reported only if the study did not state the planned energy of prescribed diets.

‡‡ Trials with a MQS score ≥ 8 were considered to be of higher quality.

§§ Agency funding is that from government, university, or not-for-profit health agency sources. None of the trialists declared any conflicts of interest with the exception of Jenkins et al.[33] and Darvish Damavandi et al.[32].

|||| In this study participants randomized into the almond group were instructed to consume this dose 5 days/week.

¶¶ Mixed nuts included almonds, cashews, hazelnuts, macadamia nuts, peanuts, pecans, pistachios, walnuts.

*** 43% of the participants were obese and wished to lose weight; although this was not a weight loss study, they were given advice on portion size and fat intake to help them meet their weight-reduction objective.

††† Data for this study was limited since the study’s conferences abstract and correspondence with the authors were the only sources of available data.
