# Supplementary figures and images for: Effect of Tree Nuts on Glycemic Control in Diabetes: A Systematic Review and Meta-Analysis of Randomized Controlled Dietary Trials
Source: PLoS One. 2014 Jul 30;9(7):e103376. doi: 10.1371/journal.pone.0103376 (PMC4116170; doi:10.1371/journal.pone.0103376)

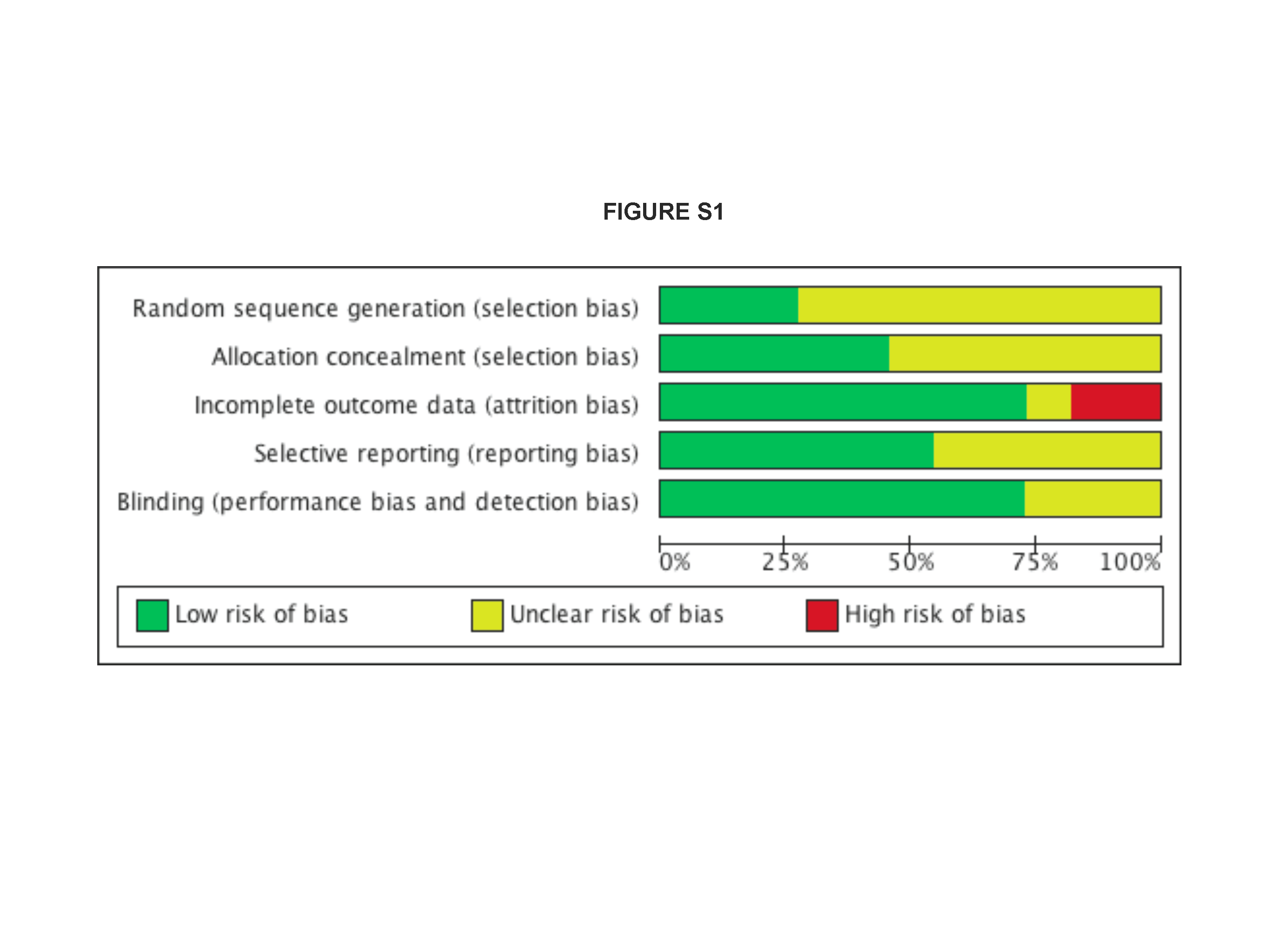

Supplement: Figure S1 — Cochrane Risk of Bias Graph. Risk of bias graph: review authors’ judgments about each risk of bias item presented as percentages across all included studies (with the exception of Sauder et al. [29]). (TIFF) [file pone.0103376.s001.tiff]

# FIGURE S2

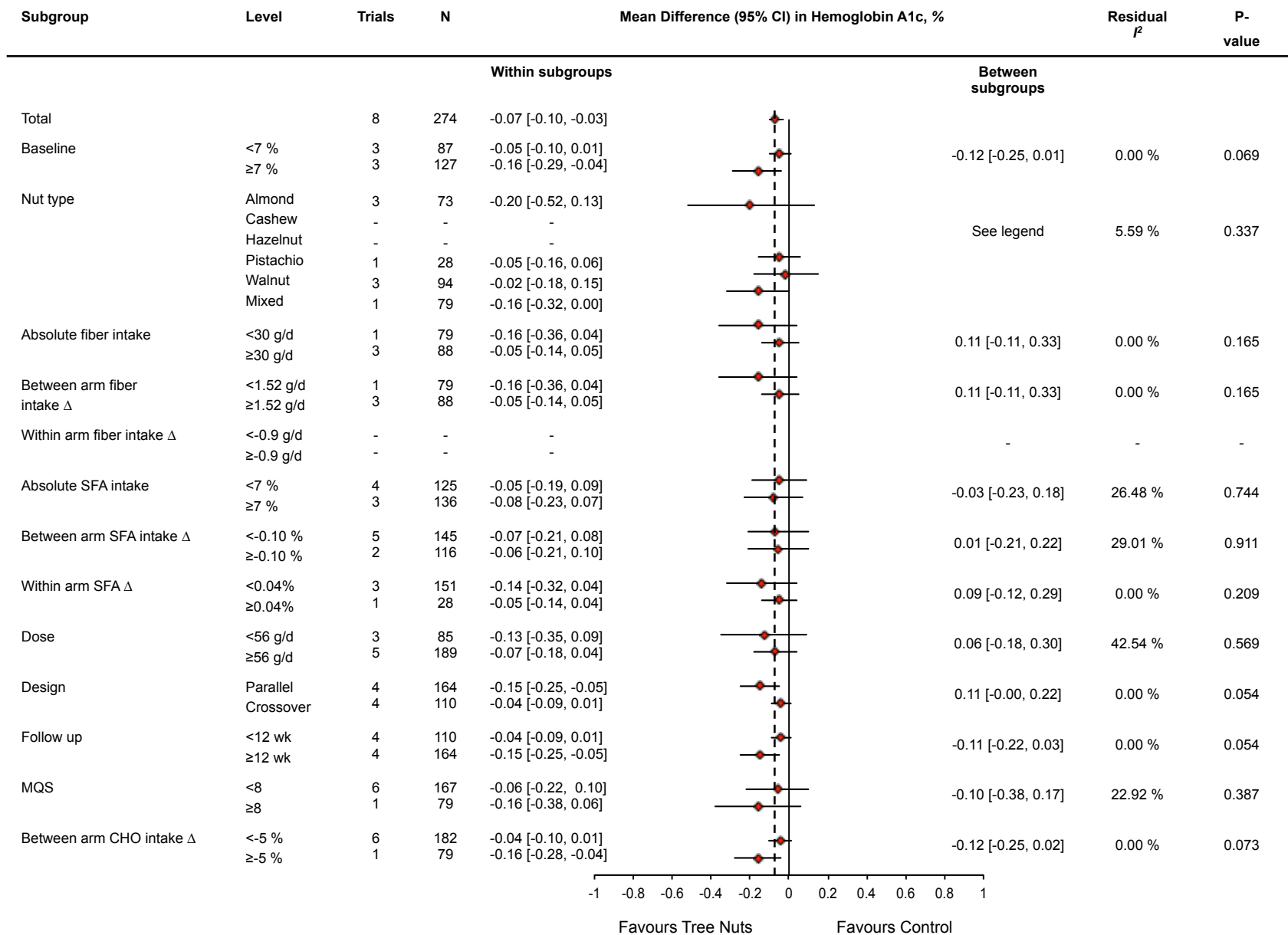

Supplement: Figure S2 — Categorical a priori and post-hoc subgroup analyses for HbA1c. CHO = carbohydrate; N = number of subjects; MQS = Heyland Methodological Quality Score; SFA = saturated fatty acid. Point estimates for each subgroup level (diamonds) are the pooled effect estimates. The dashed line represents the pooled estimate for the overall (total) analysis. The residual I2 value indicates heterogeneity unexplained by the subgroup. Pairwise between-subgroup mean differences (95%CIs) for nut type were as follows: 0.15 [−0.20, 0.49] (1 vs. 4); 0.18 [−0.18, 0.55] (1 vs. 5); 0.04 [−0.33, 0.40] (1 vs. 6); −0.03 [−0.23, 0.16] (4 vs. 5); 0.11 [−0.09, 0.31] (4 vs. 6); 0.14 [−0.09, 0.37] (5 vs. 6). Absolute intakes represent intakes within the treatment arm. Between arm differences represent the difference between the treatment (T) and control (C) arm (T–C). Within arm differences represent the difference between end (E) and baseline (B) values within the treatment arm (E–B). *Statistically significant between subgroups (P<0.05). (PDF) [file pone.0103376.s002.pdf]

FIGURE S3

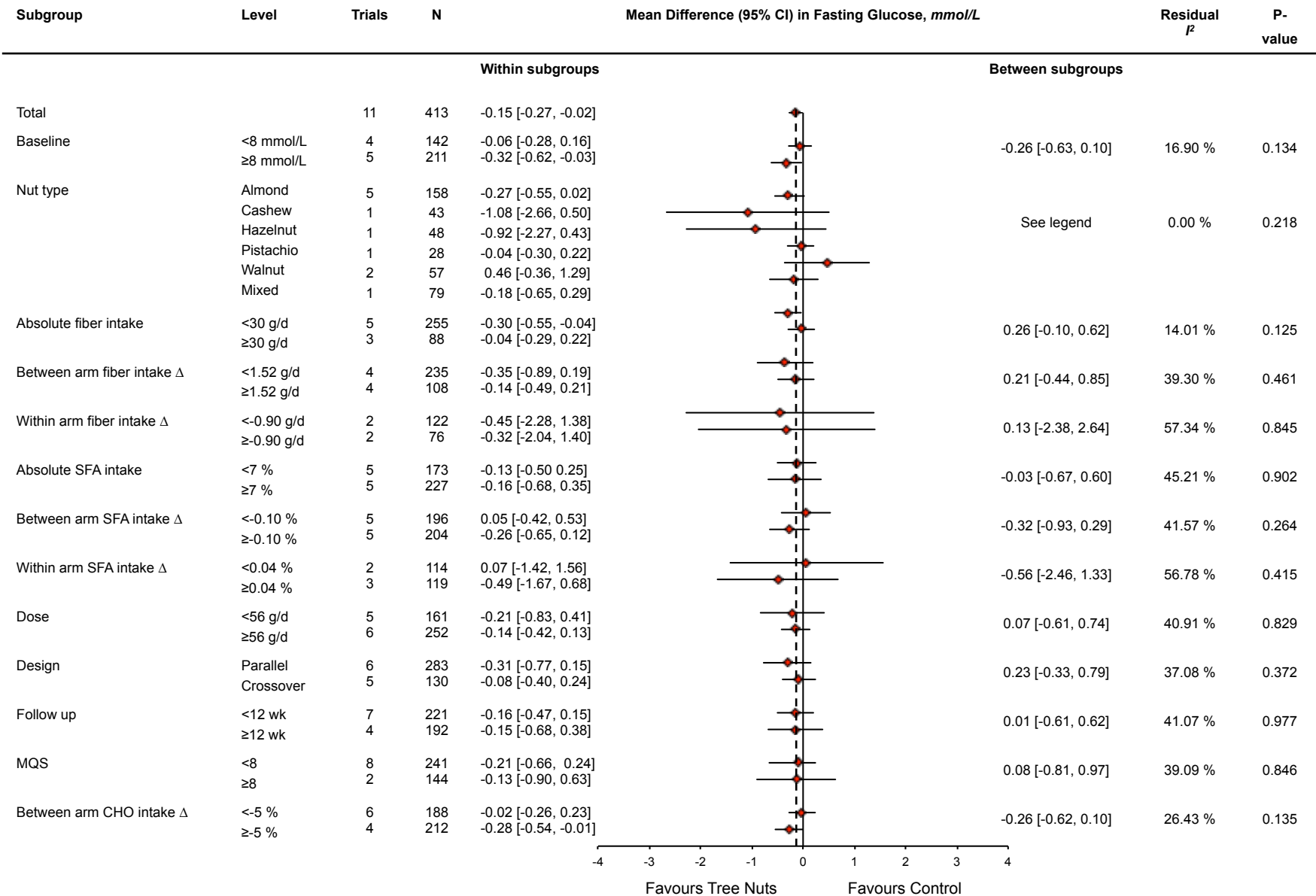

Supplement: Figure S3 — Categorical a priori and post-hoc subgroup analyses for fasting glucose. CHO = carbohydrate; N = number of subjects; MQS = Heyland Methodological Quality Score; SFA = saturated fatty acid. Point estimates for each subgroup level (diamonds) are the pooled effect estimates. The dashed line represents the pooled estimate for the overall (total) analysis. The residual I2 value indicates heterogeneity unexplained by the subgroup. Pairwise between-subgroup mean differences (95%CIs) for nut type were as follows: −0.81 [−2.41, 0.79] (1 vs. 2); −0.65 [−2.03, 0.73] (1 vs. 3); 0.23 [−0.15, 0.61] (1 vs. 4); 0.73 [−0.14, 1.60] (1 vs. 5); 0.09 [−0.46, 0.64] (1 vs. 6); −0.16 [−2.23, 1.91] (2 vs. 3); −1.04 [−2.64, 0.56] (2 vs. 4); −1.54 [−3.32, 0.23] (2 vs. 5); −0.90 [−2.55, 0.75] (2 vs. 6); −0.88 [−2.25, 0.49] (3 vs. 4); −1.38 [−2.97, 0.20] (3 vs. 5); −0.74 [−2.17, 0.69] (3 vs. 6); −0.50 [−1.37, 0.36] (4 vs. 5); 0.14 [−0.40, 0.68] (4 vs. 6); 0.64 [−0.31, 1.59] (5 vs. 6). Absolute intakes represent intakes within the treatment arm. Between arm differences represent the difference between the treatment (T) and control (C) arm (T–C). Within arm differences represent the difference between end (E) and baseline (B) values within the treatment arm (E–B). *Statistically significant between subgroups (P<0.05). (PDF) [file pone.0103376.s003.pdf]

FIGURE S4

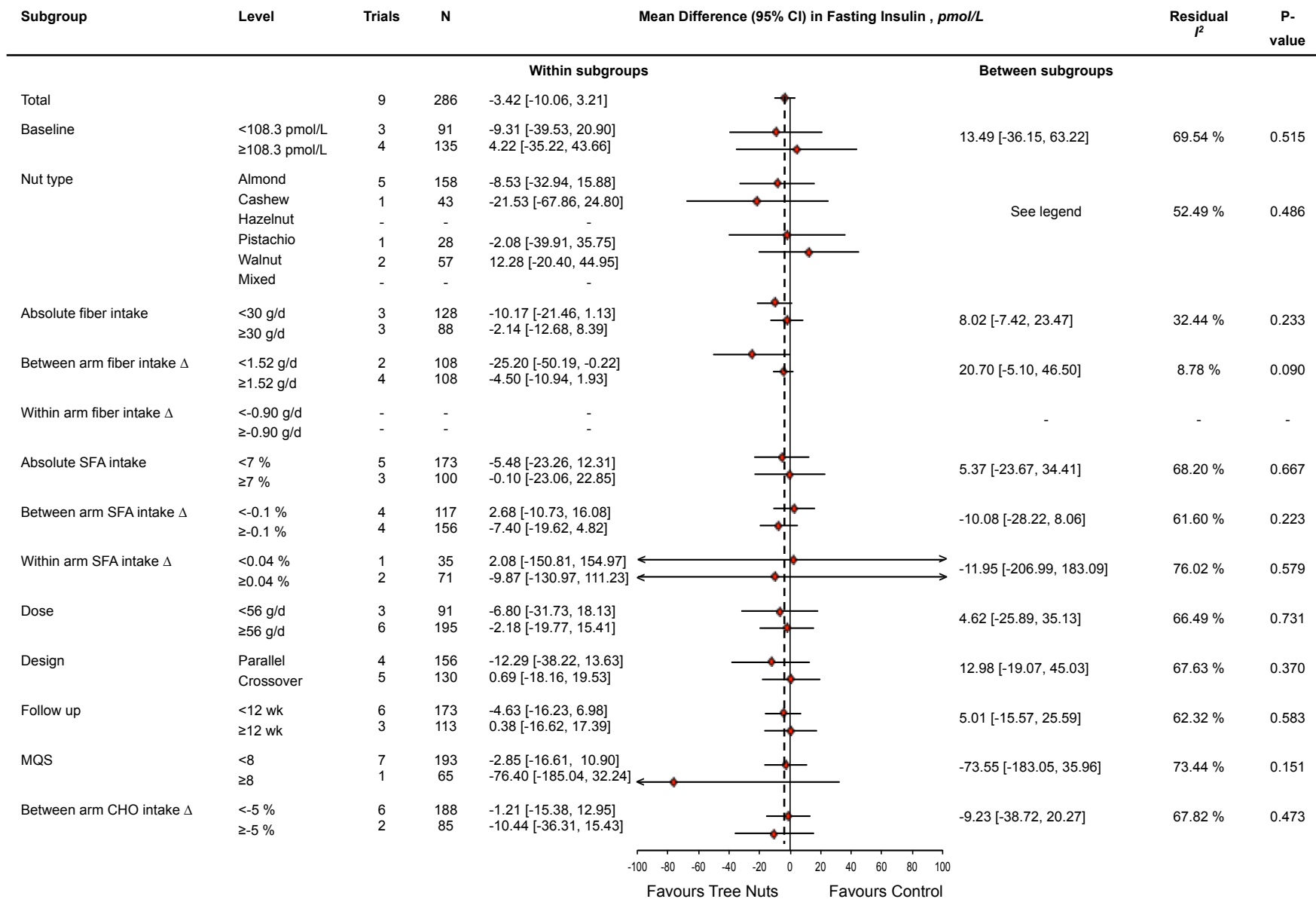

Supplement: Figure S4 — Categorical a priori and post-hoc subgroup analyses for fasting insulin. CHO = carbohydrate; N = number of subjects; MQS = Heyland Methodological Quality Score; SFA = saturated fatty acid. Point estimates for each subgroup level (diamonds) are the pooled effect estimates. The dashed line represents the pooled estimate for the overall (total) analysis. The residual I2 value indicates heterogeneity unexplained by the subgroup. Pairwise between-subgroup mean differences (95%CIs) for nut type were as follows: −13.00 [−65.37, 39.37] (1 vs. 2); 6.45 [−38.57, 51.47] (1 vs. 4); 20.81 [−19.98, 61.59] (1 vs. 5); −19.45 [−79.26, 40.36] (2 vs. 4); −31.81 [−90.50, 22.88] (2 vs. 5); −14.36 [−64.34, 35.63] (4 vs. 5). Absolute intakes represent intakes within the treatment arm. Between arm differences represent the difference between the treatment (T) and control (C) arm (T–C). Within arm differences represent the difference between end (E) and baseline (B) values within the treatment arm (E–B). * Statistically significant between subgroups (P<0.05). (PDF) [file pone.0103376.s004.pdf]

**FIGURE S5**

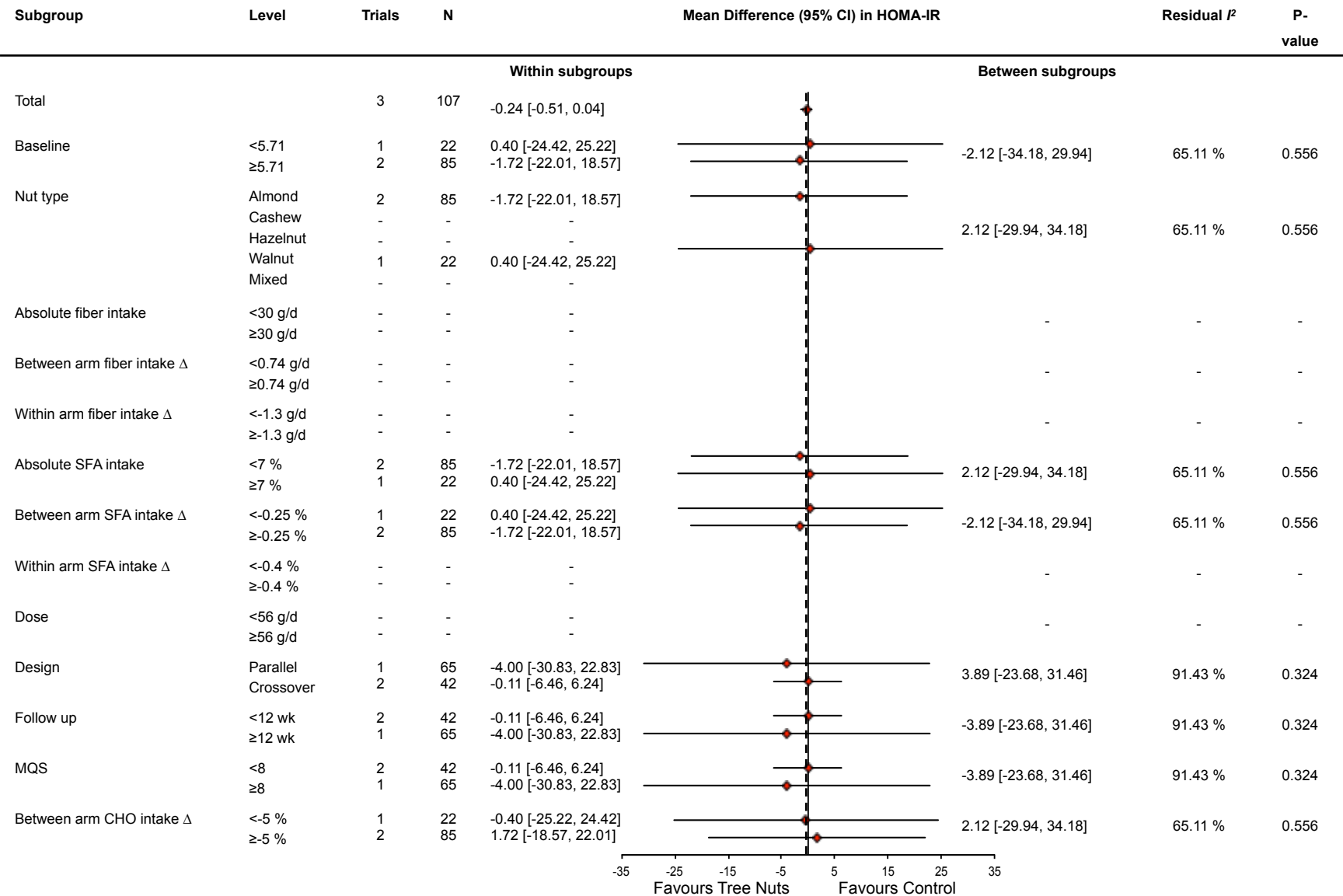

Supplement: Figure S5 — Categorical a priori and post-hoc subgroup analyses for HOMA-IR. CHO = carbohydrate; N = number of subjects; MQS = Heyland Methodological Quality Score; SFA = saturated fatty acid. Point estimates for each subgroup level (diamonds) are the pooled effect estimates. The dashed line represents the pooled estimate for the overall (total) analysis. The residual I2 value indicates heterogeneity unexplained by the subgroup. Absolute intakes represent intakes within the treatment arm. Between arm differences represent the difference between the treatment (T) and control (C) arm (T–C). Within arm differences represent the difference between end (E) and baseline (B) values within the treatment arm (E–B). * Statistically significant between subgroups (P<0.05). (PDF) [file pone.0103376.s005.pdf]
