# Supplementary material for: Effect of Tree Nuts on Glycemic Control in Diabetes: A Systematic Review and Meta-Analysis of Randomized Controlled Dietary Trials
Source: PLoS One. 2014 Jul 30;9(7):e103376. doi: 10.1371/journal.pone.0103376 (PMC4116170; doi:10.1371/journal.pone.0103376)
Supplement: Table S1 — Search strategy. For all databases, the original search was 23 May 2012; updated searches were performed 14 May 2013 and 6 April 2014. (DOCX) [file pone.0103376.s006.docx]

**TABLE S1**

| **Database** | **Search period** | **Search terms** |
| --- | --- | --- |
| MEDLINE | 1946 to April 2014 | 1. OGTT.MP. |
|  |  | 2. exp Hemoglobin A, Glycosylated/ |
|  |  | 3. hba1c.mp. |
|  |  | 4. fructosamine*.mp. |
|  |  | 5. insulin*.mp. |
|  |  | 6. glycemia.mp. |
|  |  | 7. exp Glucose/ |
|  |  | 8. exp Hyperglycemia/ |
|  |  | 9. hyperinsulin*.mp. |
|  |  | 10. dysglycemia.mp. |
|  |  | 11. gly* albumin.mp. |
|  |  | 12. exp Diabetes Mellitus/ |
|  |  | 13. metabolic syndrome.mp. |
|  |  | 14. HOMA*.mp. |
|  |  | 15. 1 or 2 or 3 or 4 or 5 or 6 or 7 or 8 or 9 or 10 or 11 or 12 or 13 or 14 |
|  |  | 16. nuts.mp. |
|  |  | 17. nut.mp. |
|  |  | 18. brazil nut.mp. |
|  |  | 19. brazil nuts.mp. |
|  |  | 20. pine nut.mp. |
|  |  | 21. pine nuts.mp. |
|  |  | 22. walnut*.mp. |
|  |  | 23. almond*.mp. |
|  |  | 24. pecan*.mp. |
|  |  | 25. pistachio*.mp. |
|  |  | 26. cashew*.mp. |
|  |  | 27. hazelnut*.mp. |
|  |  | 28. macadamia.mp. |
|  |  | 29. 16 or 17 or 18 or 19 or 20 or 21 or 22 or 23 or 24 or 25 or 26 or 27 or 28 |
|  |  | 30. 15 and 29 |
|  |  | 31. limit 30 to animals |
|  |  | 32. 30 not 31 |
| EMBASE | 1947 to April 2014 | 1. exp oral glucose tolerance test/ |
|  |  | 2. OGTT.mp. |
|  |  | 3. exp hemoglobin A1c/ |
|  |  | 4. hba1c.mp. |
|  |  | 5. fructosamine*.mp. |
|  |  | 6. insulin*.mp. |
|  |  | 7. exp glucose blood level/ |
|  |  | 8. glycemia.mp. |
|  |  | 9. exp glucose/ |
|  |  | 10. “impaired fasting glucose”.mp. |
|  |  | 11. hyperglycemia.mp. |
|  |  | 12. “impaired glucose tolerance”.mp. |
|  |  | 13. hyperinsulin*.mp. |
|  |  | 14. dysglycemia.mp. |
|  |  | 15. “gly* albumin”.mp. |

**TABLE S1 –** *Continued*

| **Database** | **Search period** | **Search terms** |
| --- | --- | --- |
| EMBASE | 1947 to April 2014 | 16. exp diabetes mellitus/ |
|  |  | 17. exp insulin dependent diabetes mellitus/ |
|  |  | 18. exp non insulin dependent diabetes mellitus/ |
|  |  | 19. exp pregnancy diabetes mellitus/ |
|  |  | 20. exp metabolic syndrome X/ |
|  |  | 21. HOMA*.mp. |
|  |  | 22. 1 or 2 or 3 or 4 or 5 or 6 or 7 or 8 or 9 or 10 or 11 or 12 or 13 or 14 or 15 or 16 or 17 or 18 or 19 or 20 or 21 |
|  |  | 23. exp nut/ |
|  |  | 24. nuts.mp. |
|  |  | 25. almond*.mp. |
|  |  | 26. walnut*.mp. |
|  |  | 27. pecan*.mp. |
|  |  | 28. pistachio*.mp. |
|  |  | 29. hazelnut*.mp. |
|  |  | 30. macadamia.mp. |
|  |  | 31. cashew*.mp. |
|  |  | 32. pine nut.mp. |
|  |  | 33. pine nuts.mp. |
|  |  | 34. brazil nut.mp. |
|  |  | 35. brazil nuts.mp. |
|  |  | 36. 23 or 24 or 25 or 26 or 27 or 28 or 29 or 30 or 31 or 32 or 33 or 34 or 35 |
|  |  | 37. 22 and 36 |
|  |  | 38. limit 37 to animals |
|  |  | 39. 37 not 38 |
| CINAHL | 1985 to 6 April 2014 | 1. OGTT |
|  |  | 2. hba1c |
|  |  | 3. (MH “Hemoglobin A, Glycosylated”) |
|  |  | 4. fructosamine* |
|  |  | 5. insulin* |
|  |  | 6. glycemia |
|  |  | 7. (MH “Glucose”) |
|  |  | 8. fasting blood glucose |
|  |  | 9. (MH “Blood Glucose”) |
|  |  | 10. (MH “Hyperglycemia+”) |
|  |  | 11. hyperinsulin* |
|  |  | 12. dysglycemia |
|  |  | 13. gly* albumin |
|  |  | 14. (MH “Diabetes Mellitus+”) |
|  |  | 15. (MH “Prediabetic State”) |
|  |  | 16. prediabetes |
|  |  | 17. (MH “Metabolic Syndrome X+”) |
|  |  | 18. metabolic syndrome |
|  |  | 19. HOMA* |
|  |  | 20. 1 or 2 or 3 or 4 or 5 or 6 or 7 or 8 or 9 or 10 or 11 or 12 or 13 or 14 or 15  or 16 or 17 or 18 or 19 |
|  |  | 21. (MH “Nuts+”) |
|  |  | 22. nut |
|  |  | 23. almond* |
|  |  | 24. walnut* |
|  |  | 25. pecan* |

**TABLE S1 –** *Continued*

| **Database** | **Search period** | **Search terms** |
| --- | --- | --- |
| CINAHL | 1985 to 6 April 2014 | 26. pistachio* |
|  |  | 27. cashew* |
|  |  | 28. hazelnut* |
|  |  | 29. macadamia* |
|  |  | 30. brazil nut |
|  |  | 31. brazil nuts |
|  |  | 32. pine nut |
|  |  | 33. pine nuts |
|  |  | 34. 22 or 23 or 24 or 25 or 26 or 27 or 28 or 29 or 30 or 31 or 32 or 33 |
|  |  | 35. 20 and 34 |
| The Cochrane Central Register of Controlled Trials | through to 6 April 2014 | 1.OGTT.mp. |
|  |  | 2. “oral glucose tolerance test”.mp. |
|  |  | 3. exp Hemoglobin A, Glycosylated/ |
|  |  | 4. hba1c.mp. |
|  |  | 5. fructosamine*.mp. |
|  |  | 6. insulin*.mp. |
|  |  | 7. glycemia.mp. |
|  |  | 8. exp Glucose/ |
|  |  | 9. exp Hyperglycemia/ |
|  |  | 10. hyperinsulin*.mp. |
|  |  | 11. dysglycemia.mp. |
|  |  | 12. gly* albumin.mp. |
|  |  | 13. exp diabetes mellitus/ |
|  |  | 14. metabolic syndrome.mp. |
|  |  | 15. HOMA*.mp. |
|  |  | 16. 1 or 2 or 3 or 4 or 5 or 6 or 7 or 8 or 9 or 10 or 11 or 12 or 13 or 14 or 15 |
|  |  | 17. nuts.mp. |
|  |  | 18. nut.mp. |
|  |  | 19. walnut*.mp. |
|  |  | 20. almond*.mp. |
|  |  | 21. pistachio*.mp. |
|  |  | 22. brazil nut.mp. |
|  |  | 23. brazil nuts.mp. |
|  |  | 24. hazelnut*.mp. |
|  |  | 25. macadamia.mp. |
|  |  | 26. pine nut.mp. |
|  |  | 27. pine nuts.mp. |
|  |  | 28. pecan*.mp. |
|  |  | 29. cashew*.mp. |
|  |  | 30. 17 or 18 or 19 or 20 or 21 or 22 or 23 or 24 or 25 or 26 or 27 or 28 or 29 |
|  |  | 31. 16 and 30 |
