# Supplementary material for: Effect of Tree Nuts on Glycemic Control in Diabetes: A Systematic Review and Meta-Analysis of Randomized Controlled Dietary Trials
Source: PLoS One. 2014 Jul 30;9(7):e103376. doi: 10.1371/journal.pone.0103376 (PMC4116170; doi:10.1371/journal.pone.0103376)
Supplement: Table S2 — Study Quality Assessment using the Heyland MQS*. HF = high fat diet; LF = low fat diet; MQS = Methodological Quality Score. * The Heyland MQS assigns a score of 0 or 1 or from 0 to 2 over 9 categories of quality related to study design, sampling procedures, and interventions for a total of 13 points. Trials that scored ≥8 were considered to be of higher quality [25]. † Randomization was scored 2 points for being randomized with the methods described, 1 point for being randomized without the methods described, or 0 points for being neither randomized nor having the methods described. Blinding was scored 1 point for being double-blind or 0 points for “other.” Analysis was scored 2 points for being intention-to-treat; all other types of analyses scored 0 points. ‡ Sample selection was scored 1 point for being consecutive eligible or 0 points for being preselected or indeterminate. Sample comparability was scored 1 point for being comparable or 0 points for not being comparable at baseline. Follow-up was scored 1 point for being 100% or 0 points for <100%. § Treatment protocol was scored 1 point for being reproducibly described or 0 points for being poorly described. Co-interventions were scored 2 points for being described and equal, 1 point for being described but unequal or indeterminate, or 0 points for not being described. Treatment crossovers (where participants were switched from the control treatment to the experimental treatment) were scored 2 points for being <10%, 1 point for being >10%, and 0 points for not being described. || Study quality for this study was not assessed since data for this study was limited (the study’s conferences abstract and correspondence with the authors were the only sources of available data). (DOCX) [file pone.0103376.s007.docx]

**TABLE S2**

| **Study, Year (Reference)** | **Design**† | | | | **Sample**‡ | | | **Intervention**§ | | **MQS**  **(n/13)** |
| --- | --- | --- | --- | --- | --- | --- | --- | --- | --- | --- |
|  | **Randomization (n/2)** | **Blinding (n/1)** | **Analysis (n/2)** | **Selection (n/1)** | **Compatibility (n/1)** | **Follow-up (n/1)** | **Protocol (n/1)** | **Co-interventions (n/2)** | **Crossovers (n/2)** |  |
| Lovejoy et al, 2002-HF ([27](#_ENREF_21)) | 1 | 1 | 0 | 0 | 1 | 0 | 0 | 2 | 0 | 5 |
| Lovejoy et al, 2002-LF ([27](#_ENREF_21)) | 1 | 1 | 0 | 0 | 1 | 0 | 0 | 2 | 0 | 5 |
| Wien et al, 2003 ([28](#_ENREF_22)) | 2 | 0 | 2 | 0 | 1 | 0 | 1 | 2 | 0 | 8 |
| Tapsell et al, 2004 (37) | 1 | 0 | 2 | 1 | 1 | 0 | 0 | 1 | 0 | 6 |
| Tapsell et al, 2009 (36) | 2 | 0 | 0 | 1 | 1 | 0 | 1 | 2 | 0 | 7 |
| Ma et al, 2010 (35) | 1 | 0 | 0 | 1 | 1 | 0 | 1 | 1 | 0 | 5 |
| Cohen et al, 2011 (30) | 1 | 0 | 2 | 0 | 1 | 1 | 0 | 2 | 0 | 7 |
| Jenkins et al, 2011 (33) | 1 | 0 | 2 | 1 | 1 | 0 | 1 | 2 | 0 | 8 |
| Li et al, 2011 ([34](#_ENREF_26)) | 1 | 0 | 0 | 0 | 1 | 0 | 1 | 2 | 0 | 5 |
| Darvish Damavandi et al, 2012 ([32](#_ENREF_24)) | 1 | 0 | 0 | 0 | 0 | 0 | 1 | 1 | 0 | 3 |
| Darvish Damavandi et al, 2013 ([38](#_ENREF_30)) | 1 | 0 | 0 | 0 | 1 | 0 | 1 | 1 | 0 | 4 |
| Sauder et al, 2013 (29) \|\| | - | - | - | - | - | - | - | - | - | - |
