# Supplementary material for: Effect of Tree Nuts on Glycemic Control in Diabetes: A Systematic Review and Meta-Analysis of Randomized Controlled Dietary Trials
Source: PLoS One. 2014 Jul 30;9(7):e103376. doi: 10.1371/journal.pone.0103376 (PMC4116170; doi:10.1371/journal.pone.0103376)
Supplement: Table S6 — Continuous a priori and post-hoc subgroup analyses for HOMA-IR. BMI = body mass index; CHO = carbohydrate; E = energy; N = number of subjects; No. = number; SFA = saturated fatty acid. β is the slope derived from subgroup analyses on meta-regression analyses and represents the treatment effect of tree nuts for each subgroup. The residual I2 value indicates heterogeneity unexplained by the subgroup. Absolute intakes represent intakes within the treatment arm. Between arm differences represent the difference between the treatment (T) and control (C) arm (T–C). Within arm differences represent the difference between end (E) and baseline(B) values within the treatment arm (E–B). * Statistically significant between subgroups (P<0.05). (DOCX) [file pone.0103376.s011.docx]

**TABLE S6**

| **Subgroup category** | **Range** | **No. of Trials (#/3)** | **N** | **β (95% CI)** | **Residual I^2^ (%)** | **P-value** |
| --- | --- | --- | --- | --- | --- | --- |
| Dose | 56-84 g/d | 3 | 107 | -0.14 (-1.12, 0.85) | 91.43 | 0.324 |
| Absolute fiber intake | - | - | - | - | - | - |
| Between arm fiber intake ∆ | - | - | - | - | - | - |
| Within arm fiber intake ∆ | - | - | - | - | - | - |
| Absolute SFA intake | 3-10.9 % E | 3 | 107 | 0.36 (-2.58, 3.30) | 45.68 | 0.362 |
| Between arm SFA intake ∆ | -1.6-0 % E | 3 | 107 | -1.29 (-20.79, 18.21) | 65.11 | 0.556 |
| Within arm SFA intake ∆ | - | - | - | - | - | - |
| Between arm CHO intake ∆ | -21- -4 % E | 3 | 107 | 0.18 (-0.41, 0.77) | 0.00 | 0.163 |
| BMI | 26-39 kg/m^2^ | 3 | 107 | -0.21 (-3.56, 3.15) | 85.44 | 0.580 |
| Sex | - | - | - | - | - | - |
