# Supplementary material for: Effect of Tree Nuts on Glycemic Control in Diabetes: A Systematic Review and Meta-Analysis of Randomized Controlled Dietary Trials
Source: PLoS One. 2014 Jul 30;9(7):e103376. doi: 10.1371/journal.pone.0103376 (PMC4116170; doi:10.1371/journal.pone.0103376)
Supplement: Protocol S1 — Trial protocol. (PDF) [file pone.0103376.s013.pdf]

ClinicalTrials.gov
A service of the U.S. National Institutes of Health

Meta-analyses of the Effect of Tree Nuts on Glycemic Control and Features of the Metabolic Syndrome

This study is ongoing, but not recruiting participants.

Sponsor:
John Sievenpiper

Collaborators:
The International Tree Nut Council Nutrition Research & Education Foundation
Canada Research Chairs Endowment of the Federal Government of Canada
Canadian Institutes of Health Research (CIHR)

Information provided by (Responsible Party):
John Sievenpiper, University of Toronto

ClinicalTrials.gov Identifier:
NCT01630980

First received: May 18, 2012
Last updated: January 6, 2014
Last verified: July 2013
History of Changes

Tracking Information	
**First Received Date** ICMJE	May 18, 2012
**Last Updated Date**	January 6, 2014
**Start Date** ICMJE	May 2012
**Estimated Primary Completion Date**	March 2014 (final data collection date for primary outcome measure)
**Current Primary Outcome Measures** ICMJE **(submitted: June 26, 2012)**	• Glycemic control measures [Time Frame: Up to 1.5-years] [Designated as safety issue: No]<br>Glycated blood proteins (HbA1c, total glycated hemoglobin, fructosamine, glycated albumin), fasting glucose, fasting insulin, and the homeostasis model assessment of insulin resistance (HOMA-IR)<br>• Metabolic syndrome measures [Time Frame: Up to 1.5-years] [Designated as safety issue: No]<br>Harmonized metabolic syndrome diagnostic measures (waist circumference, TG, HDL-C, blood pressure, fasting glucose)
**Original Primary Outcome Measures** ICMJE	*Same as current*
**Change History**	Complete list of historical versions of study NCT01630980 on ClinicalTrials.gov Archive Site
**Current Secondary Outcome Measures** ICMJE	*Not Provided*
**Original Secondary Outcome Measures** ICMJE	*Not Provided*
**Current Other Outcome Measures** ICMJE	*Not Provided*
**Original Other Outcome Measures** ICMJE	*Not Provided*

Descriptive Information

**Brief Title** ICMJE	Meta-analyses of the Effect of Tree Nuts on Glycemic Control and Features of the Metabolic Syndrome
**Official Title** ICMJE	Effect of Tree Nuts on Glycemic Control and Features of the Metabolic Syndrome: A Systematic Review and Meta-analysis of Controlled Dietary Trials to Provide Evidence-based Guidance for Nutrition Guidelines Development
**Brief Summary**	Tree nuts (almonds, Brazil nuts, cashews, hazelnuts, macadamia nuts, pecans, pine nuts, pistachios and walnuts) are an important source of unsaturated fatty acids, vegetable protein, and fibre, as well as minerals, vitamins, and phytonutrients. Although heart disease risk reduction claims for nuts have been permitted in the U.S. and general dietary guidelines and recommendations from heart associations recommend the consumption of nuts for heart protection, diabetes associations have not addressed nuts in their most recent recommendations. This omission is despite heart disease being a major cause of death in diabetes. There remains insufficient information on the usefulness of these foods in diabetes. To improve evidence-based guidance for tree nut recommendations, the investigators propose to conduct a systematic review of the effect of tree nuts on diabetes control and features of the metabolic syndrome. The systematic review process allows the combining of the results from many small studies in order to arrive at a pooled estimate, similar to a weighted average, of the true effect. The investigators will be able to explore whether eating tree nuts has different effects between men and women, in different age groups and background disease states, and whether or not the effect of tree nuts depends on the dose and background diet. The findings of this proposed knowledge synthesis will help improve the health of Canadians through informing diabetes association recommendations and heart association recommendations where they relate to diabetes.
**Detailed Description**	Background: Tree nuts (almonds, Brazil nuts, cashews, hazelnuts, macadamia nuts, pecans, pine nuts,<br><br>pistachios and walnuts)are an important source of unsaturated fatty acids, vegetable protein, and fibre, as well as minerals, vitamins, and phytonutrients. Although the dietary guidelines for Americans and American Heart Association (AHA) recommend the consumption of nuts for cardiovascular risk reduction and the US Food and Drug Administration (FDA) issued a qualified coronary heart disease (CHD) risk reduction claim for nuts, none of the diabetes associations have addressed nuts in their most recent recommendations. This omission is despite CHD being a major cause of death in diabetes. Several trials have been undertaken in diabetes, some of which, including the largest to date by our group, have demonstrated advantages in glycemic control. Although the remaining trials have failed to show a significant improvement in glycemic control, the direction of the effect has favored nuts, along with improvements in complementary markers of metabolic control.<br><br>Need for a review: The lack of high quality data in this area to support diabetes recommendations represents an urgent call for stronger evidence. A systematic review and meta-analysis of controlled feeding trials remains the "Gold Standard" of evidence for nutrition guidelines development.<br><br>Objective: To provide evidence-based guidance for diabetes guidelines, we will conduct two systematic reviews and meta-analyses of controlled feeding trials to assess the effect of tree nuts (almonds, Brazil nuts, cashews, hazelnuts, macadamia nuts, pecans, pine nuts, pistachios and walnuts) on cardiometabolic control: (1) "Tree nuts and glycemic control" and (2) "Tree nuts and features of the metabolic syndrome".<br><br>Design: The planning and conduct of the proposed meta-analyses will follow the Cochrane handbook for systematic reviews of interventions. The reporting will follow the Preferred Reporting Items for Systematic reviews and Meta-Analyses (PRISMA) guidelines.<br><br>Data sources: MEDLINE, EMBASE, CINAHL and The Cochrane Central Register of Controlled Trials will be searched using appropriate search terms.<br><br>Study selection: Intervention trials that investigate the effect of exchanging nuts for other diets on cardiometabolic risk outcomes in humans will be included. Studies that are <3-weeks diet duration, lack a control, or report non-isocaloric comparisons will be excluded.<br><br>Data extraction: Independent investigators (≥2) will extract information about study design, sample size, subject characteristics, nut form, dose, follow-up, and the composition of the background diets. Mean±SEM values will be extracted for all endpoints. Standard computations and imputations will be used to derive missing variance data. Risk of bias and study quality will be assessed using the risk of bias tool and the Heyland Methodological Quality Score (MQS), respectively.<br><br>Outcomes: The two proposed analyses will assess a set of outcomes related to a different area of cardiometabolic control: (1) glycemic control (glycated blood proteins[HbA1c, fructosamine, glycated albumin], fasting glucose, fasting insulin, and the homeostasis model assessment of insulin resistance [HOMA-IR]) and (2) harmonized metabolic syndrome features (waist circumference, TG, HDL-C, blood pressure, fasting glucose).

	harmonized metabolic syndrome features (waist circumference, TG, HDL-C, blood pressure, fasting glucose).<br><br>Data synthesis: Pooled analyses will be conducted using the Generic Inverse Variance method with random effects models. Random-effects models will be used even in the absence of statistically significant between-study heterogeneity, as they yield more conservative summary effect estimates in the presence of residual heterogeneity. Exceptions will be made for the use of fixed-effects models where there is <5 included trials or small trials are being pooled with larger more precise trials in the absence of statistically significant between-study heterogeneity. Paired analyses will be applied to all crossover trials. Heterogeneity will be tested by Cochrane's Q and quantified by I2. Sources of heterogeneity will be explored by sensitivity and subgroup analyses. A priori subgroup analyses will include nut type, nut dose, duration of follow-up, change in saturated fat intake, change in dietary fibre intake, design (crossover, parallel), study quality, and baseline endpoint values. Significant unexplained heterogeneity will be investigated by additional post hoc subgroup analyses (e.g.<br><br>age, sex, level of feeding control [metabolic, supplemented, dietary advice], washout in crossover trials, energy balance of the background diet, composition of the background diet [total % energy from fat, carbohydrate, protein], change in cholesterol intake, change in glycemic index, etc.). Meta-regression analyses will assess the significance of subgroups analyses. Publication bias will be investigated by the inspection of funnel plots and application of Egger's and Begg's tests.<br><br>Knowledge translation plan: Results from the two systematic reviews and meta-analyses will be disseminated through traditional means such as interactive presentations at local, national, and international scientific meetings and publication in high impact factor journals. Innovative means such as webcasts with e-mail feedback mechanisms will also be used. Knowledge Users will act as knowledge brokers networking among opinion leaders and different adopter groups to increase awareness at each stage. Two of the applicants (JLS, CWCK) will also participate directly as members of nutrition guidelines committees the 2013 CDA Clinical Practice Guidelines (CPG) for nutrition therapy by one of the applicants (JLS) and 2015 European Association for the Study of Diabetes (EASD) CPG for nutrition therapy (JLS, CWCK). Target adopters will include the clinical practice, public health, industry, research communities, and patient groups. Feedback will be incorporated and used to guide analyses and improve key messages at each stage.<br><br>Significance: The two proposed systematic reviews and meta-analyses will aid in knowledge translation related to the effects of tree nuts in diabetes and metabolic syndrome, strengthening the evidence-base for dietary recommendations and health claims.
**Study Type** ICMJE	Observational
**Study Design** ICMJE	Time Perspective: Prospective
**Target Follow-Up Duration**	*Not Provided*
**Biospecimen**	*Not Provided*
**Sampling Method**	Probability Sample
**Study Population**	Varied
**Condition** ICMJE	• Diabetes<br>• Prediabetes<br>• Dysglycemia<br>• Overweight<br>• Obesity<br>• Dyslipidemia<br>• Hypertension<br>• Metabolic Syndrome<br>• Cardiovascular Disease Risk
**Intervention** ICMJE	Other: Tree nuts<br>Almonds, Brazil nuts, cashews, hazelnuts, macadamia nuts, pecans, pine nuts, pistachios and walnuts in whole, meal, or flour form
**Study Group/Cohort (s)**	*Not Provided*
**Publications** *	Jenkins DJ, Kendall CW, Banach MS, Srichaikul K, Vidgen E, Mitchell S, Parker T, Nishi S, Bashyam B, de

Souza R, Ireland C, Josse RG. Nuts as a replacement for carbohydrates in the diabetic diet. Diabetes Care. 2011 Aug;34(8):1706-11. Epub 2011 Jun 29.

*** Includes publications given by the data provider as well as publications identified by ClinicalTrials.gov Identifier (NCT Number) in Medline.**

Recruitment Information

**Recruitment Status** ICMJE	Active, not recruiting
**Estimated Enrollment** ICMJE	1
**Estimated Completion Date**	March 2014
**Estimated Primary Completion Date**	March 2014 (final data collection date for primary outcome measure)
**Eligibility Criteria** ICMJE	Inclusion Criteria:<br>• Dietary trials in humans<br>• Randomized treatment allocation<br>• >=3-weeks<br>• Suitable control (i.e. isocaloric exchange of other dietary components for tree nuts)<br>• viable endpoint data<br>Exclusion Criteria:<br>• Non-human studies<br>• Nonrandomized treatment allocation<br>• 3-weeks<br>• Lack of a suitable control (non-isocaloric)<br>• no viable endpoint data
**Gender**	Both
**Ages**	*Not Provided*
**Accepts Healthy Volunteers**	No
**Contacts** ICMJE	*Contact information is only displayed when the study is recruiting subjects*
**Location Countries** ICMJE	Canada

Administrative Information

**NCT Number** ICMJE	NCT01630980
**Other Study ID Numbers** ICMJE	INC 2012 KRS
**Has Data Monitoring Committee**	No
**Responsible Party**	John Sievenpiper, University of Toronto
**Study Sponsor** ICMJE	John Sievenpiper
**Collaborators** ICMJE	• The International Tree Nut Council Nutrition Research & Education Foundation<br>• Canada Research Chairs Endowment of the Federal Government of Canada

	• Canadian Institutes of Health Research (CIHR)
**Investigators** ICMJE	Study Director: John L Sievenpiper, MD, PhD — Department of Pathology and Molecular Medicine, McMaster University and Clinical Nutrition and Risk Factor Modification Centre, St. Michael's Hospital<br>Study Director: Russell J de Souza, ScD, RD — Department of Epidemiology and Biostatistics, McMaster University and Clinical Nutrition and Risk Factor Modification Centre, St. Michael's Hospital<br>Principal Investigator: Cyril WC Kendall, PhD — Department of Nutritional Sciences and Medicine, University of Toronto and Clinical Nutrition and Risk Factor Modification Centre, St. Michael's Hospital<br>Principal Investigator: David JA Jenkins, MD, PhD, DSc — Department of Nutritional Sciences and Medicine, University of Toronto and Clinical Nutrition and Risk Factor Modification Centre, St. Michael's Hospital
**Information Provided By**	University of Toronto
**Verification Date**	July 2013

ICMJE **Data element required by the International Committee of Medical Journal Editors and the World Health Organization ICTRP**
